# Fluoride responsive single nanochannel: click fabrication and highly selective sensing in aqueous solution†
†Electronic supplementary information (ESI) available. See DOI: 10.1039/c5sc02191j


**DOI:** 10.1039/c5sc02191j

**Published:** 2015-07-22

**Authors:** Guanrong Nie, Yue Sun, Fan Zhang, Miaomiao Song, Demei Tian, Lei Jiang, Haibing Li

**Affiliations:** a Key Laboratory of Pesticide and Chemical Biology (CCNU) , Ministry of Education , College of Chemistry , Central China Normal University , Wuhan 430079 , P. R. China . Email: lhbing@mail.ccnu.edu.cn; b Beijing National Laboratory for Molecular Sciences (BNLMS) , Key Laboratory of Organic Solids , Institute of Chemistry , Chinese Academy of Sciences , Beijing , 100190 , P. R. China

## Abstract

Fluoride is important to explore because it plays important roles in nature and biological processes. To accomplish selective F^–^ sensing in aqueous solution, a nanochannel modified with 1,3-dipropargylaza-*p-tert*-butyl calix[4]crown (C4CE) was designed on the basis of hydrogen-bonding interactions. The nanodevice not only exhibits high selectivity for F^–^, even in serum, but also shows the useful property of recyclability.

## Abstract 2

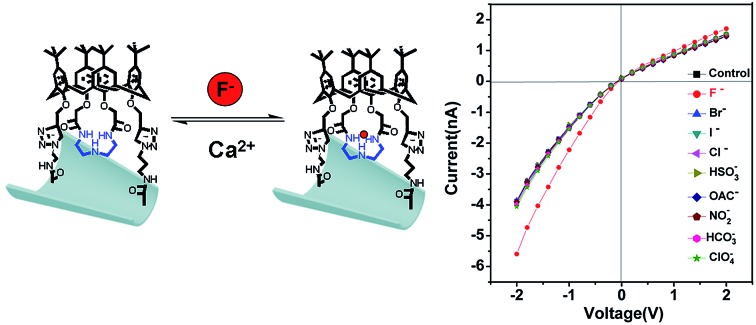
A F^–^ responsive nanochannel based on hydrogen-bonding interactions was designed to accomplish highly selective sensing in aqueous solution.

## Introduction

Anions exist widely in living organisms and play essential roles in nature and biological processes.^1^ Among anions, F^–^ is of great importance to our health because of its duplicitous nature.^2^ As is known to all, fluorine is a required trace element in the human body. Appropriate levels of fluorine have an important effect on the formation of the skeleton and teeth, and thus can prevent dental caries and senior osteoporosis.^3^ However, an excess of F^–^ accumulated in the body can cause a number of diseases, such as skeletal fluorosis,^4^ kidney failure^5^ and nephrolithiasis.^6^ Therefore, it is very meaningful to develop a simple, sensitive and rapid approach for F^–^ sensing. Over the past decade, fluorescent chemosensors for fluoride have been developed rapidly. Most of the probes, however, were used for F^–^ sensing in organic solution^7^ or mixed organic–aqueous solution,^8^ and also suffered from interference with other anions, which greatly limits the scope of their application. Hence, a device for F^–^ sensing with the advantages of simplicity and high selectivity in aqueous solution, especially in complicated biological samples, is still absent and urgently needed.

In living cells, ion channels on cell membranes can selectively transport specific ions. Inspired by biological channels, solid-state synthetic nanochannels have been developed, owing to the fact that they possess good mechanical and chemical stability.^9^,^10^ In recent years, artificial nanochannels have been widely employed as simple sensing devices because they realize highly sensitive detection^11^ by directly measuring transmembrane current without signal transfer apparatus. Thus, a series of artificial nanochannels were fabricated to respond to bio-macromolecules with large size, such as DNA,^12^ proteins,^13^*etc.*, which result in distinct changes in transmembrane current in confined nanochannels. To the best of our knowledge, anion chemosensors based on artificial nanochannels have been largely unexplored. This may be ascribed to the small size of the anions, especially F^–^, and strong solvent effects in water. Hence, it is a challenging task to develop single artificial nanochannels for F^–^ sensing in aqueous solution.

**Scheme 1:**
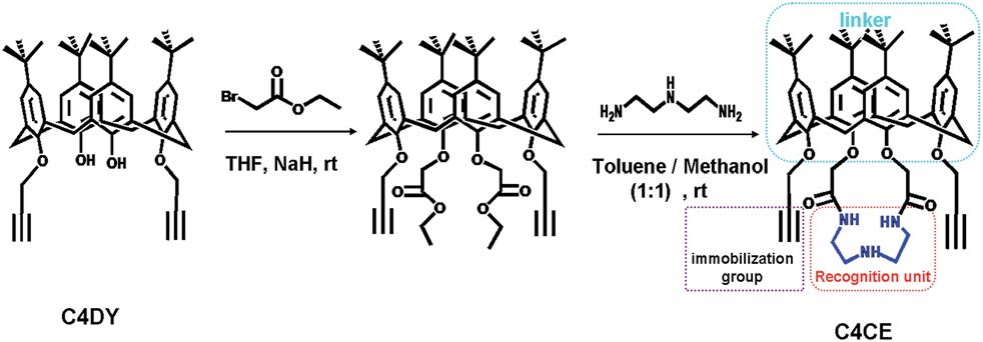
The synthesis route of C4CE designed for F^–^ sensing.

To address this challenging task, we developed a new strategy of introducing host–guest interactions onto the internal wall of the nanochannel to achieve a selective response to F^–^. Accordingly, it is key to design an appropriate host for F^–^. To the best of our knowledge, the –NH– functional group can be selectively combined with fluoride by hydrogen-bonding interactions. To weaken the competition effect from H_2_O, *N*-(2-aminoethyl)ethane-1,2-diamine with three hydrogen-bonding donors was chosen as the recognition unit and was expected to form a cluster of hydrogen-bonding donors to reinforce the binding interaction. On the other hand, to solve the problem of the immobilization of the recognition unit on the nanochannel, an easily derived calix[4]arene was chosen as the platform to link the immobilized group and the recognition unit. Based on this, we used 1,3-dipropylalkynyl calix[4]arene (C4DY) as the starting material to design and synthesize 1,3-dipropargylaza-*p-tert*-butyl calix[4]crown (C4CE), in which the alkynyl group served as the immobilized group to fix the C4CE on the inner surface of the single nanochannel and the –NH– groups were used as the recognition unit for F^–^ sensing, by a two-step reaction as shown in Scheme 1. Fortunately, the target molecule C4CE was synthesized successfully and was characterized by ^1^H NMR, ^13^C NMR, and MS (ESI†).

## Experimental section

### Reagents and materials

Polyimide (PI, 12 μm thick) membranes were irradiated with heavy ions at the UNILAC linear accelerator (GSI, Darmstadt, Germany). 1-Ethyl-3-(3-dimethylaminopropyl)carbodiimide (EDC), potassium iodide (KI), *N*-hydroxysulfosuccinimide (NHS), sodium hypochlorite (NaClO), 4-morpholine ethyl sulfonic acid (MES), sodium ascorbate, copper sulfate pentahydrate (CuSO_4_·5H_2_O), potassium chloride (KCl), sodium bromide (NaBr), sodium iodide (NaI), sodium chloride (NaCl), sodium hydrogen sulfite (NaHSO_3_), sodium acetate (NaOAC), sodium nitrate (NaNO_2_), sodium hydrogen carbonate (NaHCO_3_), sodium perchlorate (NaClO_4_), and tris(hydroxymethyl)aminomethane hydrochloride (Tris–HCl) were purchased from Sinopharm Chemical Reagent Shanghai Co., Ltd (SCRC, China). All solutions were prepared in MilliQ water (18.2 MΩ).

### Fabrication of the single conical nanochannel

The single conical nanochannel was prepared in PI film by the ion track-etching technique. In order to obtain the conical nanochannel, the etching was performed in only one side of the conductivity cell, and the other side of the cell was filled with stopping solution which was able to neutralize the etchant as soon as the pore opened. In this work, the PI membrane was embedded between the two chambers of a conductivity cell at 50 °C. One chamber was filled with etching solution (NaClO, 13% available chlorine, pH ≈ 12.5), and the other chamber was filled with stopping solution (1 M KI). Then, a voltage of 1 V was applied across the membrane to monitor the current. The etching process was stopped at a desired current value corresponding to a certain tip diameter. The membrane was immersed in MilliQ water (18.2 MΩ) to remove residual salts. The diameter of the large opening of the conical nanochannel which was called the base (*D*), was determined by scanning electron microscopy (SEM), and the diameter of the small opening which was called the tip (*d*_tip_) was estimated using the following relation:^14^
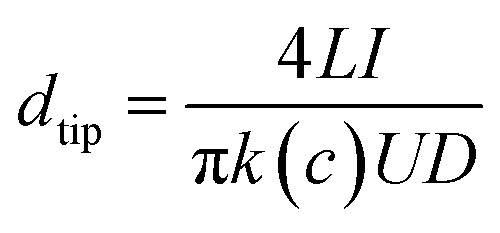


**Fig. 1:**
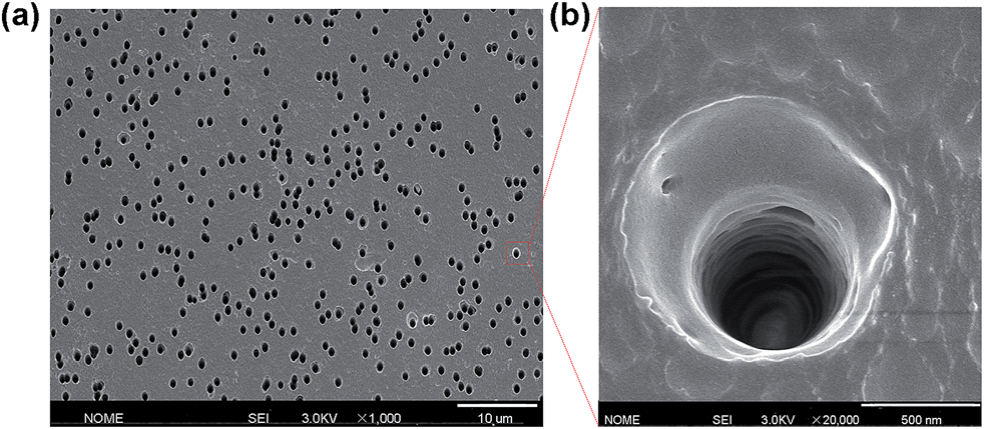
(a) Scanning electron microscopy (SEM) image in track-etched PI film; (b) the enlarged image.

*L* is the length of the pore; *I* is the current; *U* is the applied voltage; *d*_tip_ and *D* are the tip diameter and the base diameter respectively; and *k*(*c*) is the specific conductivity of the electrolyte. For 1 M KCl solution at 25 °C, *k*(*c*) is 0.11173 Ω^–1^ cm^–1^ (Fig. 1). In this work, the base diameter was about 1 μm and the tip diameter was about 15 nm.

### Modification

The nanochannel was modified using the following procedure: the first step was to immobilize 3-azidopropan-1-amine (APAM) on the nanochannel by a classical EDC/NHS coupling reaction. After the chemical etching process, carboxyl (–COOH) groups were generated on the channel surface. These could be converted into amine-reactive esters *via* carbodiimide coupling chemistry and then further reacted with APAM molecules through the formation of covalent bonds. In this work, the single conical nanochannel was immersed in an aqueous solution of 30 mg EDC and 6 mg NHS at pH 5.4 for 1 h at room temperature. After that, the film was washed with distilled water and treated with 1 mM APAM overnight. Subsequently, the APAM-modified nanochannel was washed with distilled water and was used in the next step. The second step was to fix the 1,3-dipropargylaza-*p-tert*-butyl calix[4]crown (C4CE) on the nanochannel by click chemistry of the azide group (–N_3_) and the alkynyl group (–C≡CH). The APAM-modified nanochannel was exposed to a solution of 0.087 mg mL^–1^ CuSO_4_·5H_2_O, 0.14 mg mL^–1^ sodium ascorbate and 1 mM 1,3-dipropargylaza-*p-tert*-butyl calix[4]crown (C4CE) overnight. Finally, the modified film was washed three times with distilled water.

## Results and discussion

### The ^1^H NMR and ^19^F NMR analysis of C4CE and F^–^

According to the literature,^15^ there are the following two possibilities for the interactions between –NH– groups and F^–^: the N–H···F hydrogen-bonding interaction and the deprotonation of the –NH– groups by fluoride. To confirm the true nature of the interactions in our work, we carried out ^1^H NMR analysis in DMSO and ^19^F NMR analysis in THF. As shown in Fig. S3,† the –NH– proton peak of the host C4CE at 7.64 ppm exhibited a downfield shift of about 0.03 ppm after one equivalent of F^–^ was added. In order to clearly observe the downfield shift phenomenon, ^1^H NMR titrations were carried out in DMSO. As seen in Fig. S4,† sequential addition of increasing equivalents of F^–^ ions resulted in broadening and continuous downfield shift of the –NH– proton peak at 7.64 ppm. At the same time, no HF_2_^–^ peak at 16 ppm caused by deprotonation was observed, even if 16 equivalents of F^–^ ions were added. Hence, we speculate that the interactions between the receptor C4CE and the F^–^ ions are N–H···F hydrogen-bonding interactions. To further verify this speculation, ^19^F NMR spectra were recorded in THF solvent. As shown in Fig. S5,† a new peak at 131.1 ppm appeared in the ^19^F NMR spectrum after F^–^ was added, which suggests that N–H···F was generated. This process is consistent with an earlier report.^16^ In addition, the binding interaction of –NH– and F^–^ was affected by the solvent, especially H_2_O. It is worth noting that our nanochannel system was used in 100% water. According to the earlier literature,^17^ F^–^ does not induce deprotonation and only hydrogen-bonding between the anion and receptor is detected in buffered solution containing 50% water. From the above comprehensive analysis, the interactions between the receptor C4CE and the F^–^ ions are ascribed to N–H···F hydrogen-bonding interactions.

### The construction of the C4CE channel

**Fig. 2:**
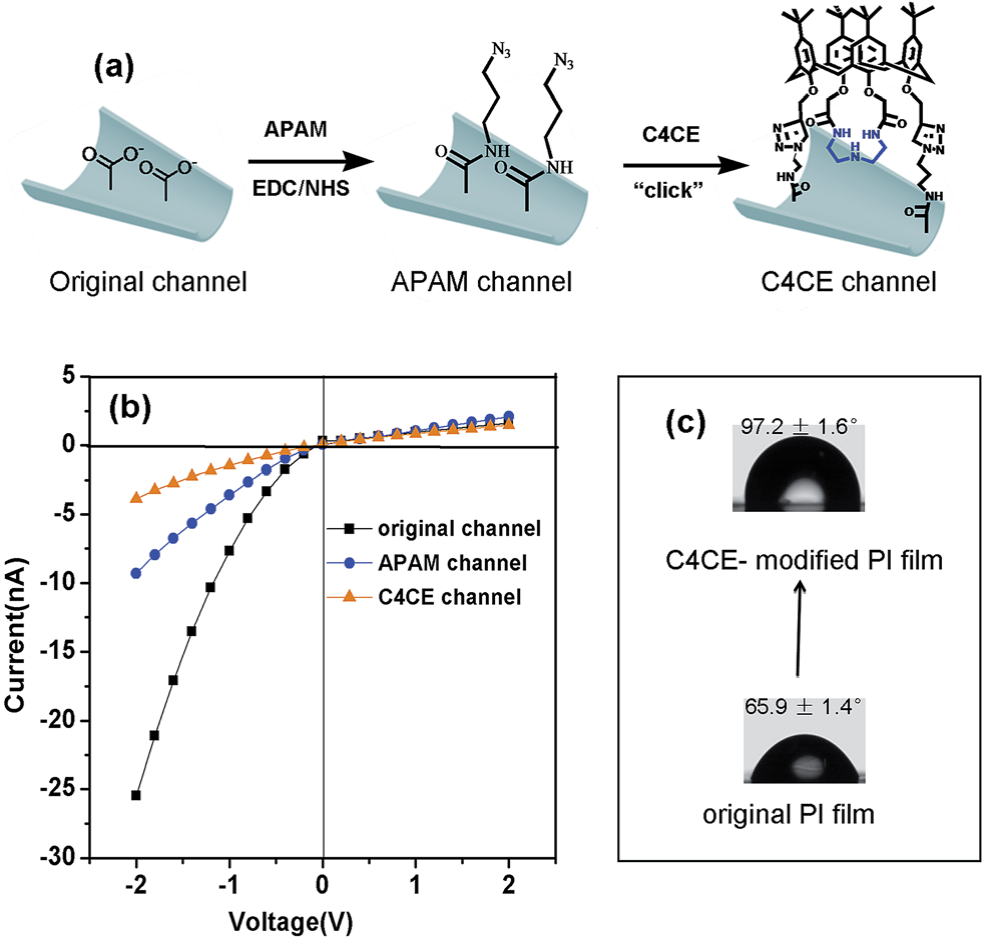
The successful fabrication of the C4CE channel. (a) The construction process of the single conical nanochannel for F^–^ sensing; (b) the changes in the *I*–*V* curve of the single nanochannel after each modification; (c) the contact angel on the flat film before and after modification of C4CE. These indicated that the C4CE host was modified successfully.

Based on the interactions between C4CE and F^–^, the fluoride-responsive nanochannel was constructed as shown in Fig. 2a. After the chemical etching process, the carboxyl groups were exposed on the internal surface of the single conical nanochannel. The 3-azidopropan-1-amine (APAM) molecules were immobilized on the nanochannel by a classical EDC/NHS coupling reaction. Further, the alkynyl group from C4CE was linked with an azide group (–N_3_), which was exposed on the surface of the nanochannel, by a click reaction. Hence, the C4CE was introduced onto the internal surface of the nanochannel by a two-step reaction. As shown in Fig. 2b, the *I*–*V* curve of the single nanochannel changed after each modification. The original conical nanochannel exhibited a sharp rectified ionic current in an electrolyte of 0.1 M KCl and 0.05 M tris(hydroxymethyl)aminomethane hydrochloride (Tris–HCl) at pH 7.0 due to the negative charge and conical shape. After the immobilization of APAM on the nanochannel, the current decreased distinctly, which could be explained by changes in the negative charge and wettability. Similarly, the current was reduced further after C4CE was immobilized on the nanochannel, which could be caused by the lack of charge and the hydrophobic effect of C4CE. To some extent, the changes in the *I*–*V* curve demonstrated successful modification of the C4CE molecules.

In order to further verify the successful modification of C4CE, contact angle measurements (seen in Fig. 2c), X-ray photoelectron spectroscopy (XPS) tests (seen in Fig. S7†) and laser scanning confocal microscopy (LSCM) tests (seen in Fig. 4a) were carried out. As shown in Fig. 2c, the wettability changed dramatically after the modification of C4CE. The contact angle of the original PI film without modification was 65.9 ± 1.4°. After APAM was immobilized on the PI film, the contact angle increased to 83.7 ± 2.8° (seen in Fig. S5†), resulting from the hydrophobicity of the APAM, which is one of the reasons for the decrease in current for the APAM-modified nanochannel, as discussed above. After the addition of C4CE to the PI film, the contact angle continued to increase to 97.2 ± 1.6° due to the hydrophobicity of C4CE, which is one of the reasons for the further decrease in current for the C4CE-modified nanochannel. For the XPS tests, in order to characterize the nitrogen from APAM and C4CE and avoid interference from the nitrogen present in the original PI film, the PI film was substituted with PET film with the same –COO^–^ groups exposed on the surface. The PET films did not contain nitrogen, and a peak due to nitrogen appeared after modification with APAM and C4CE, indicating the successful modification of C4CE by the click reaction. Subsequently, successful modification of C4CE could be further confirmed by the LSCM tests. In order to observe fluorescence after C4CE was immobilized on the nanochannel, fluorescent dansyl chloride (DNS) was introduced into the C4CE host according to a literature method.^18^ Accordingly, the fluorescent host DNS-C4CE was synthesized and immobilized on the PI multi-channel membrane. As can be seen in Fig. 4a, fluorescence could not be observed for the original channel. After the immobilization of DNS-C4CE, the nanochannel exhibited fluorescence, which indicated successful modification of the multi-channels. Hence, all of the above evidence demonstrated that the C4CE channel was successfully constructed by the click reaction.

### The F^–^ response of the C4CE channel

**Fig. 3:**
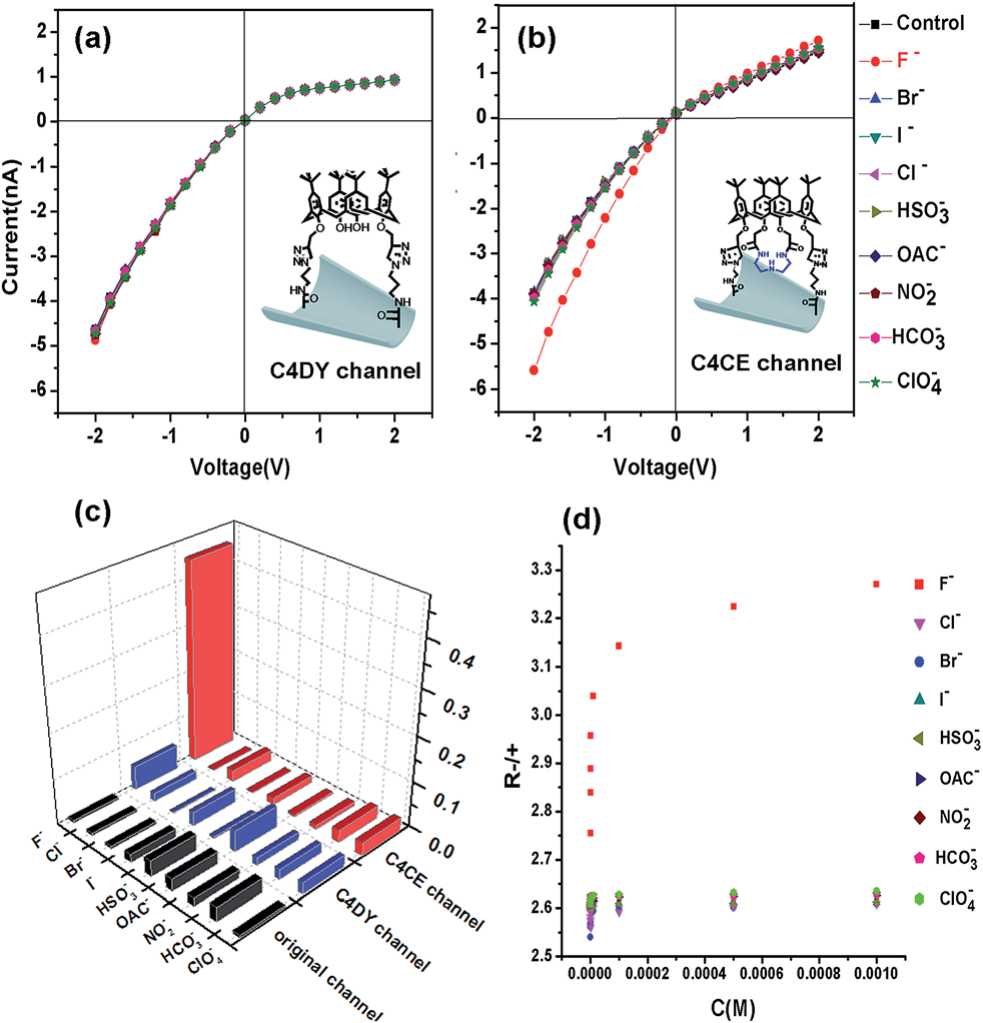
(a) The *I*–*V* curves of the nanochannel modified with C4DY; (b) *I*–*V* curves of the C4CE nanochannel in an electrolyte of 0.1 M KCl and 0.05 M Tris–HCl (pH = 7.0) in the presence of 1 mM F^–^, Cl^–^, Br^–^, I^–^, HSO_3_^–^, OAC^–^, NO_2_^–^, HCO_3_^–^, and ClO_4_^–^; (c) histogram of the ionic current change ratios ((*I* – *I*_0_)/*I*_0_) at –2 V after adding the above ions to the original channel, the C4DY channel, and the C4CE channel; (d) the change in *R*_-/+_ of the C4CE channel in the presence of different concentrations of the respective ions. These results indicated that the C4CE nanochannel was selectively responsive to F^–^.

The selective recognition performance was evaluated by measuring the ionic current across the nanochannel in 0.1 M KCl–Tris–HCl buffer solution at pH 7.0 while adding 1 mM F^–^, Cl^–^, Br^–^, I^–^, HSO_3_^–^, OAC^–^, NO_2_^–^, HCO_3_^–^ and ClO_4_^–^. As can be seen in Fig. S7,† there were no distinct changes in the ionic current observed in the presence of F^–^, Cl^–^, Br^–^, I^–^, HSO_3_^–^, OAC^–^, NO_2_^–^, HCO_3_^–^ and ClO_4_^–^ for the original and APAM channels, which suggested no ion selectivity. However, after the immobilization of C4CE, the nanochannel showed a distinct response to F^–^. As shown in Fig. 3b, a remarkable increase in the ionic current was observed when F^–^ was added to the electrolyte, while there was hardly any change in the ionic current in the presence of other ions like Cl^–^, Br^–^, I^–^, HSO_3_^–^, OAC^–^, NO_2_^–^, HCO_3_^–^ and ClO_4_^–^. In order to investigate whether the binding site for F^–^ depends on the recognition unit in C4CE, a C4DY channel was fabricated and studied under the same conditions as the C4CE channel. However, after addition of all the respective anions, the current did not show any obvious change, which demonstrated that the binding site for F^–^ depends on the recognition unit. In order to more clearly observe the high selectivity for F^–^ of the C4CE channel, a histogram of the ionic current change ratios (defined as the absolute value of the ratio of the current change at –2 V, Ratio = ∣*I* – *I*_0_∣/*I*_0_, where *I* means the current at –2 V after adding certain analyte ions, and *I*_0_ means the current at –2 V without addition of analyte ions) is depicted in Fig. 3c. It can be seen that the C4CE nanochannel exhibited a highly selective response to F^–^.

To evaluate the sensitivity to F^–^ of the C4CE channel, the change in the *I*–*V* curve of the C4CE nanochannel upon the addition of F^–^ with concentrations ranging from 10^–9^ M to 10^–3^ M was investigated. As seen in Fig. S9,† with increasing concentration of F^–^, the ionic current at –2 V gradually increased. This phenomenon could be interpreted as follows: with increasing concentration of F^–^, the number of F^–^ ions captured by the C4CE channel increases, which gives rise to an increase in the negative charge on the surface of the channel and thus causes an increase in the current. Even if the concentration of F^–^ was as low as 10^–9^ M, the ionic change was still obvious, which indicated the highly sensitive response to F^–^ of the C4CE channel. In this work, the calculated detection limit is 9.7 × 10^–7^ M (seen in Table S5†). However, the ionic current hardly changed with increasing concentrations of the other anions. To illustrate the change more clearly, the change in *R*_-/+_ is shown in Fig. 3d, where the rectification ratio (*R*_-/+_) is defined as the ratio of the absolute value of the current recorded at –2 V to the value of the current recorded at +2 V. *R*_-/+_ increased on increasing the ion concentration from 10^–9^ to 10^–3^ M, which clearly suggested the good selectivity for F^–^ of the C4CE channel.

### Confirmation of the response mechanism of the nanochannel

**Fig. 4:**
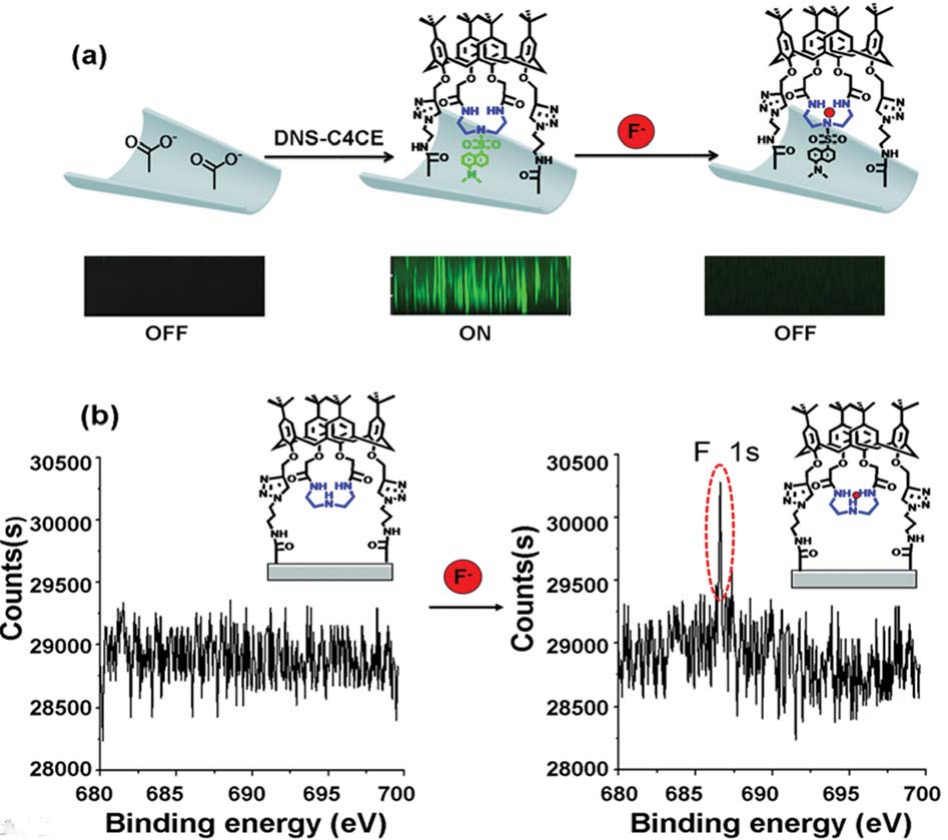
(a) Laser scanning confocal microscopy (LSCM) images observed without DNS-C4CE, with DNS-C4CE and after immersing the DNS-C4CE-modified porous PI film in 10^–3^ M F^–^ solution; (b) the XPS spectra for the C4CE-modified film before and after immersion in 10^–3^ M F^–^ solution. These confirmed that the C4CE host can capture F^–^.

The possible response mechanism is elucidated as follows: as discussed above regarding the ^1^H NMR and ^19^F NMR spectra, the selective response to F^–^ of the C4CE channel was attributed to hydrogen-bonding interactions between C4CE and F^–^. To directly confirm that the C4CE channel can actually capture F^–^, LSCM and XPS tests were conducted in the absence and presence of F^–^. As shown in Fig. 4a, fluorescence was not observed for the original multi-channel, but it could be observed for the DNS-C4CE-modified channel, resulting from the successful modification of the fluorescent host DNS-C4CE. After immersing the film in 10^–3^ M F^–^ solution, the fluorescence of the DNS-C4CE-modified multi-channel weakened dramatically. A possible explanation is that the N–H···F complex formed by hydrogen-bonding interactions could quench the fluorescence of DNS-C4CE by a photo-induced election transfer (PET) mechanism.^19^ Similarly, the evidence obtained from XPS could also prove that the C4CE can capture F^–^. As shown in Fig. 4b, the F 1s peak at 686.4 eV was non-existent for the C4CE-modified film, while this peak appeared after immersing the film in 10^–3^ M F^–^ solution, indicating that F^–^ was captured on the C4CE-modified film. The above evidence proved that the C4CE channel can indeed capture F^–^ to achieve a selective response.

The *K* values (binding constants) can explain the good selectivity for F^–^ more directly. In previous literature,^20^ it was discovered that the Langmuir model provided a perfect fit to the experimental data for nanochannels. In our work, furthermore, the variation of the rectification ratio (*R*_-/+_) with increasing concentration of F^–^, as shown in Fig. 3d, was similar to the trend of the Langmuir absorption isotherm. Consequently, we speculated that it would be possible to describe the binding of F^–^ on the internal surface of the C4CE channel using the Langmuir model. According to the principle of the Langmuir equation, an analogous equation for this nanochannel was inferred as follows. *θ* was set as the fraction of occupied sites. Naturally, 1 – *θ* denotes the fraction of available binding sites. The rate of binding of analyte ions to the surface is proportional to the concentration *C* of the analyte ion and 1 – *θ*. The binding constant is named as *K*_a_. Therefore, the rate of binding of analyte ions to the surface is expressed as:

1 *r*_a_ = *K*_a_*C*(1 – *θ*)

The rate of desorption of analyte ions from the surface is proportional to *θ*. The constant of desorption is set as *K*_d_. Accordingly, the rate of desorption of analyte ions from the surface is expressed as:

2 *r*_d_ = *K*_d_*θ*

When absorption equilibrium is reached, the rate of binding is equal to the rate of desorption.

3 *r*_a_ = *r*_d_

Furthermore, the equilibrium binding constant *K* is equal to the ratio of *K*_a_ and *K*_d_.

4 *K* = *K*_a_/*K*_d_

Hence, the relationship between *θ* and *C* was obtained as follows:

5 *θ* = (*KC*)/(1 + *KC*)

For this conical nanochannel, as more F^–^ was integrated into the C4CE channel, a greater rectification ratio (*R*_-/+_ = *I*_(–2V)_/*I*_(2V)_) was observed in the ion rectification measurements. Accordingly, we assumed that the fraction of occupied sites *θ* can be approximately expressed as:

6 *θ* = *R*_-/+_/*R*_max_

Hence, the Langmuir equation for this nanochannel was obtained.

7 *C*/*R*_-/+_ = 1/*KR*_max_ + *C*/*R*_max_

According to the data from Fig. 3d, the linear relation of *C*/*R*_-/+_ – *C* shown in Fig. S9† was observed. All of the regression values were above 0.9996, which indicates a perfect fit to the experimental data for this nanochannel. This perfect linear fit suggests that the Langmuir description of the binding of F^–^ in this nanochannel is suitable. The *K* values were calculated using the linear fit equation and are shown in Table 1.

**Table 1:** *K* values (binding constants) for the different ions calculated using the Langmuir equation. *K*_F^–^_ denotes the *K* of F^–^, and *K*_(other anion)_ is the *K* of any anion except F^–^

Anion	*K*/M^–1^	*K*_F^–^_/*K*_(other anion)_
F^–^	1.17 × 10^6^	—
Cl^–^	9.60 × 10^4^	12.2
Br^–^	1.72 × 10^5^	6.8
I^–^	1.28 × 10^5^	9.2
HSO_3_^–^	2.37 × 10^5^	4.9
OAC^–^	1.87 × 10^5^	6.3
NO_2_^–^	2.80 × 10^5^	4.2
HCO_3_^–^	2.16 × 10^5^	5.4
ClO_4_^–^	3.07 × 10^5^	3.8

The *K* values for the different ions calculated using the Langmuir equation are listed in Table 1. The *K* of the C4CE channel for F^–^ was 1.17 × 10^6^ M^–1^, which was 3.8–12.2 times bigger than the *K* values for the other anions. A bigger *K*_F^–^_/*K*_(other anion)_ ratio shows a bigger difference in binding ability between F^–^ and the other anion. The *K*_F^–^_/*K*_(other anion)_ ratio was bigger for halogen ions, which could be explained by a lack of hydrogen-bonding interactions, while the *K*_F^–^_/*K*_(other anion)_ ratio was smaller for other ions, such as OAC^–^, NO_2_^–^, HSO_3_^–^, and HCO_3_^–^, which was probably caused by weak N–H···O hydrogen-bonding interactions. As shown in Table S4 (detailed in ESI†), the error in the F^–^ sensing of the C4CE channel in the presence of interfering ions was within ±10%, which indicated that the interference from these ions was weak. To the best of our knowledge, for F^–^ sensors based on hydrogen-bonding interactions, the interference from halogen ions is small, and stronger interference is mainly due to OAC^–^,^21^ resulting from the presence of hydrogen-bonding interactions between OAC^–^ and –NH– groups. In this work, nevertheless, the C4CE channel magnified the degree of differentiation between F^–^ and OAC^–^, so that the error in F^–^ sensing in the presence of OAC^–^ was decreased to 6.9%. We speculate that the reason for the increase in the degree of differentiation between F^–^ and OAC^–^ is that the flexibility of the C4CE molecule immobilized on the internal surface of the nanochannel was decreased compared to that in solution. Therefore, the shape and size requirements for ion binding were stricter. Compared with OAC^–^, F^–^ with spherical shape and smaller size is likely to preferentially bind to the C4CE host *via* hydrogen-bonding interactions.

### The recyclability of the C4CE channel

**Fig. 5:**
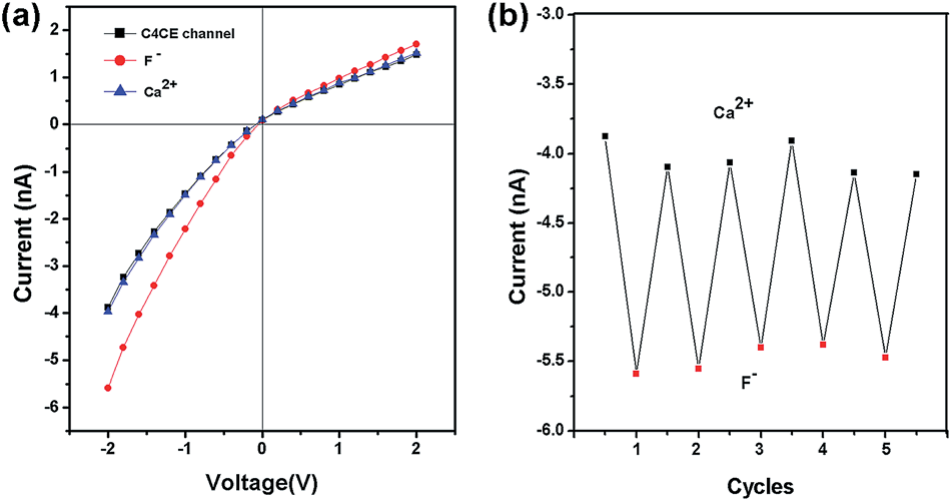
The reversibility of the C4CE nanochannel. (a) The current change for the C4CE channel on addition and removal of F^–^; (b) the recyclability of the C4CE channel shown by the current change at –2 V on addition and removal of F^–^ .

In addition, the F^–^-responsive nanodevice exhibited recyclability. The recyclability could be realized by addition and removal of F^–^. As shown in Fig. 5a, for the C4CE channel, the current increased when F^–^ was captured, while the current decreased and recovered to the low current observed in the absence of F^–^ after the nanochannel was immersed in 10^–3^ M Ca^2+^ solution, resulting from the formation of a CaF_2_ complex.^22^ As can be seen in Fig. 5b, the C4CE nanochannel exhibited good switching performance. The nanodevice can be used repeatedly, to some degree, which enhances the use ratio and economizes the cost. This is a step forward for the application of the nanodevice in real life.

### Sensing in the presence of interfering anions and serum samples

In consideration of the practical applications, the anti-interference ability was investigated in the presence of interfering anions and serum samples. In this anti-interference experiment, 10^–5^ M F^–^ and 10^–3^ M interfering ions were added to the electrolyte solution. In order to clearly reflect the anti-interference ability, the recovery rate was defined and calculated using the formula Ratio_1_/Ratio_0_, where Ratio_1_ means the current change ratio at –2 V in F^–^ solution on addition of the interfering anions, and Ratio_0_ is defined as the current change ratio at –2 V in F^–^ solution without the interfering anions. From the data shown in Table S4 (seen in ESI†), the recovery rate of F^–^ ranged from 90.2% to 109.7%, indicating that the C4CE nanochannel exhibited good anti-interference performance. Further, the system was used for F^–^ sensing in serum samples. As shown in Fig. S11,† the current for the nanochannel obviously increased in the presence of F^–^ in serum samples. Based on the current change for F^–^ sensing in the absence and presence of serum, 79.8% recovery was achieved, which showed that the C4CE nanochannel can also be used in serum samples.

## Conclusions

In summary, a novel F^–^-responsive nanodevice was successfully designed based on an artificial nanochannel modified with C4CE by a click reaction. According to the rectified ionic current measurements, this nanodevice not only exhibits high selectivity for F^–^, even in the presence of various interfering ions and serum samples, but also shows the useful property of recyclability on removal of F^–^ by Ca^2+^. It's worth noting that the nanodevice was realized by measuring the current in aqueous solution, which may be very promising for sensing and real-time monitoring, including in the environment and living organisms, and for imitating biological phenomena.

## Supplementary Material

Supplementary information

Supplementary information
