# Supplementary figures and images for: Fluoride responsive single nanochannel: click fabrication and highly selective sensing in aqueous solution
Source: Chem Sci. 2015 Jul 22;6(10):5859–65. doi: 10.1039/c5sc02191j (PMC5950555; doi:10.1039/c5sc02191j)

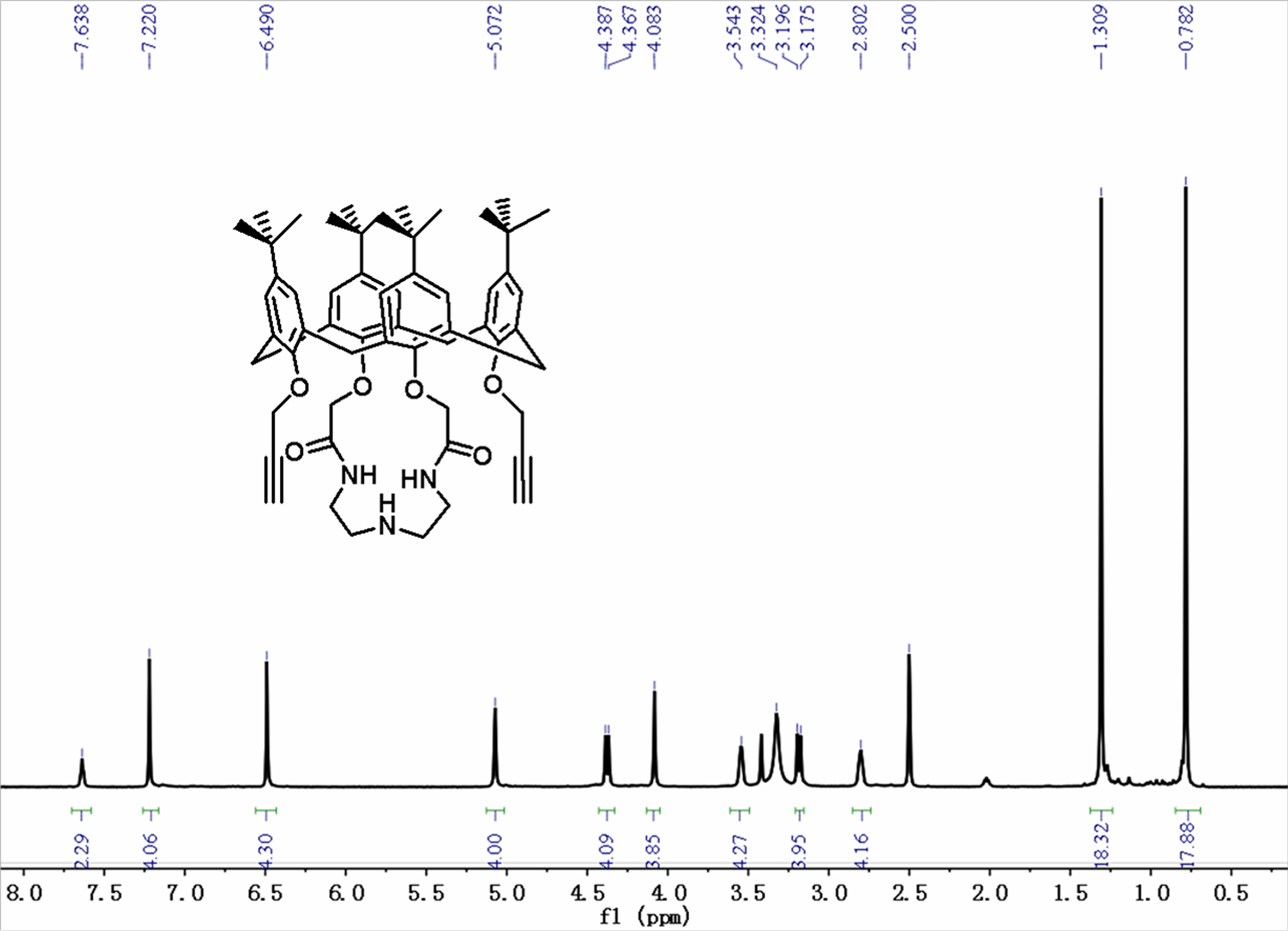

Supplement: Supplementary file 1 [file SC-006-C5SC02191J-s001.zip › fig_s1.tif]

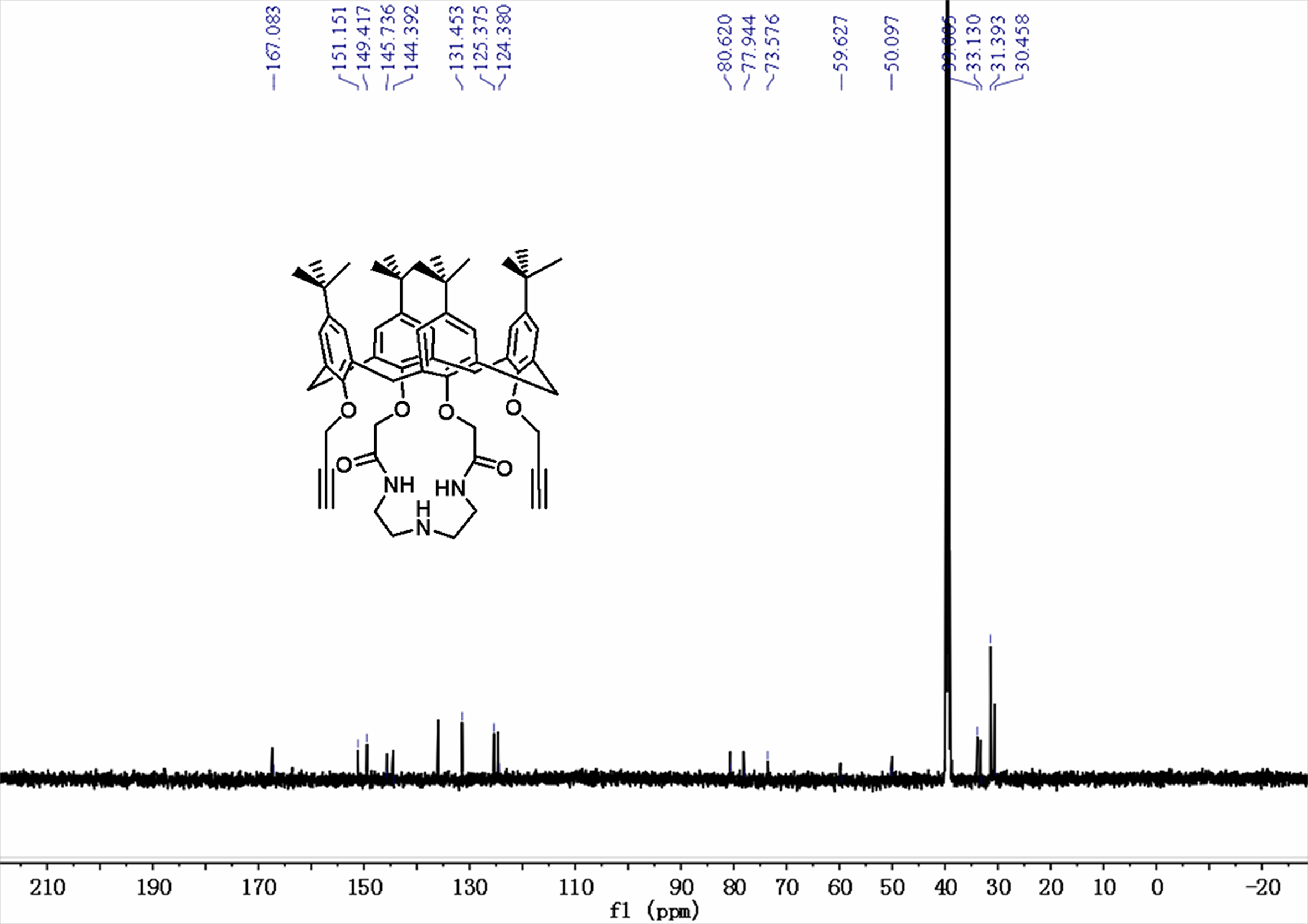

Supplement: Supplementary file 1 [file SC-006-C5SC02191J-s001.zip › fig_s2.tif]

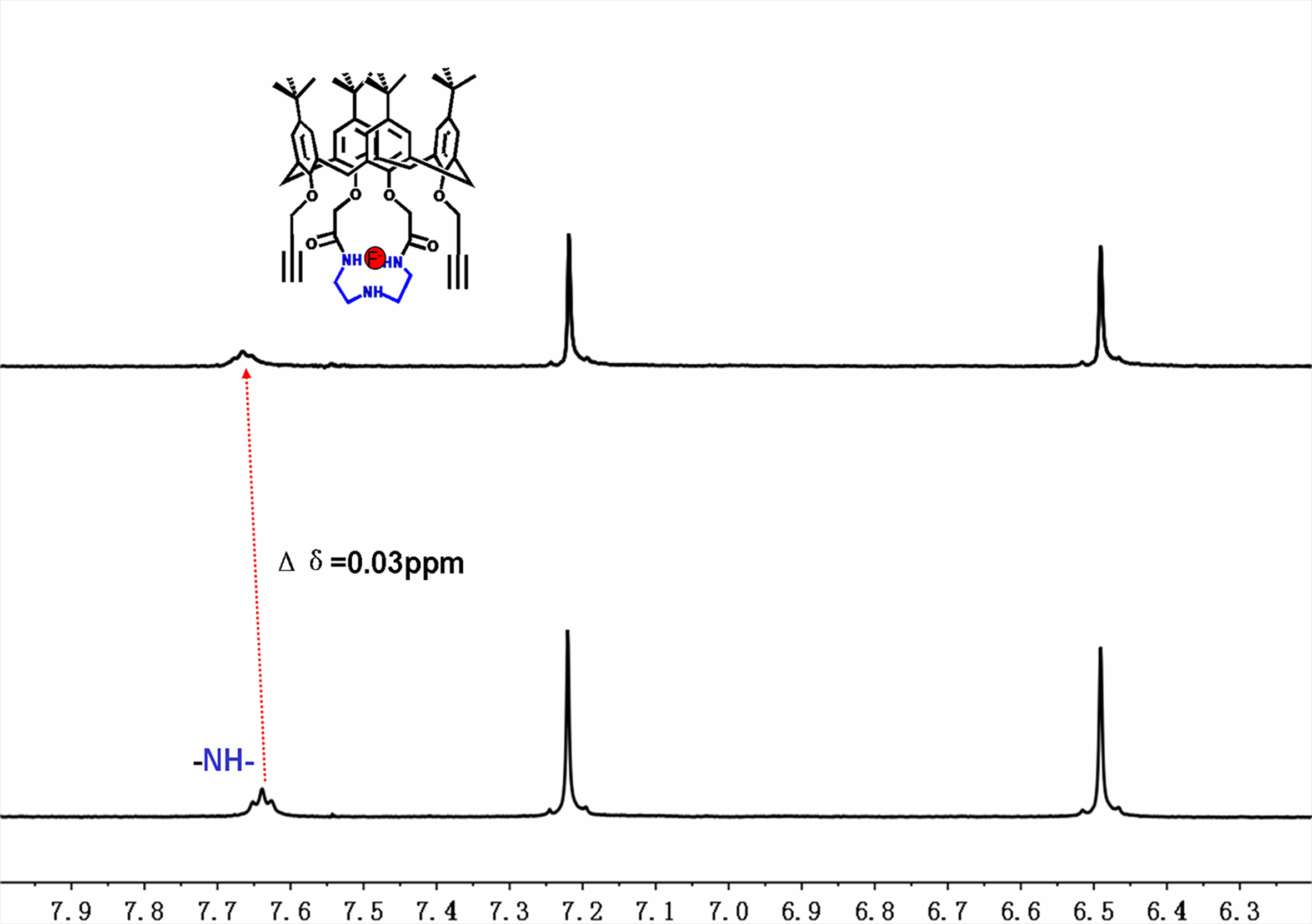

Supplement: Supplementary file 1 [file SC-006-C5SC02191J-s001.zip › fig_s3.tif]

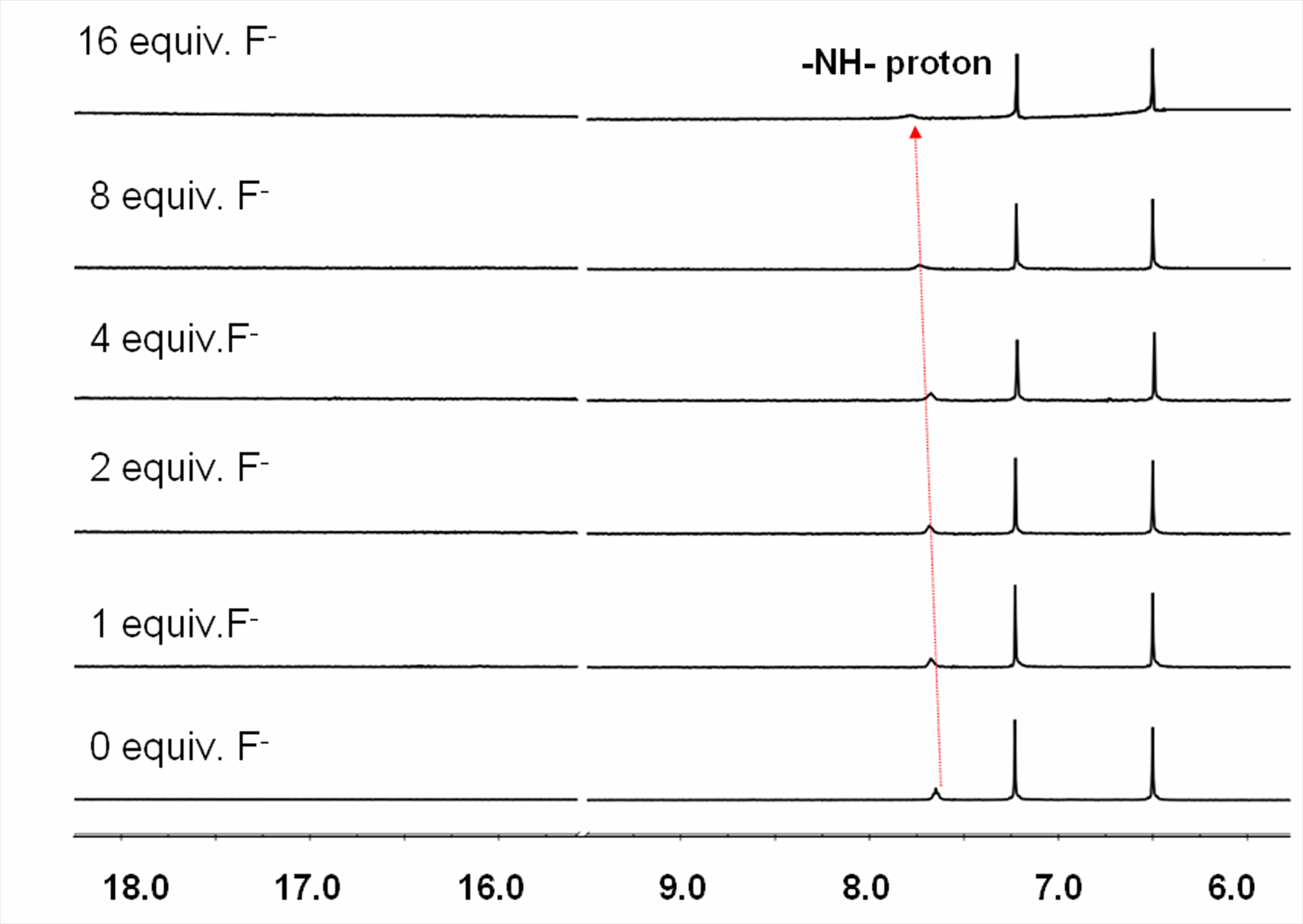

Supplement: Supplementary file 1 [file SC-006-C5SC02191J-s001.zip › fig_s4.tif]

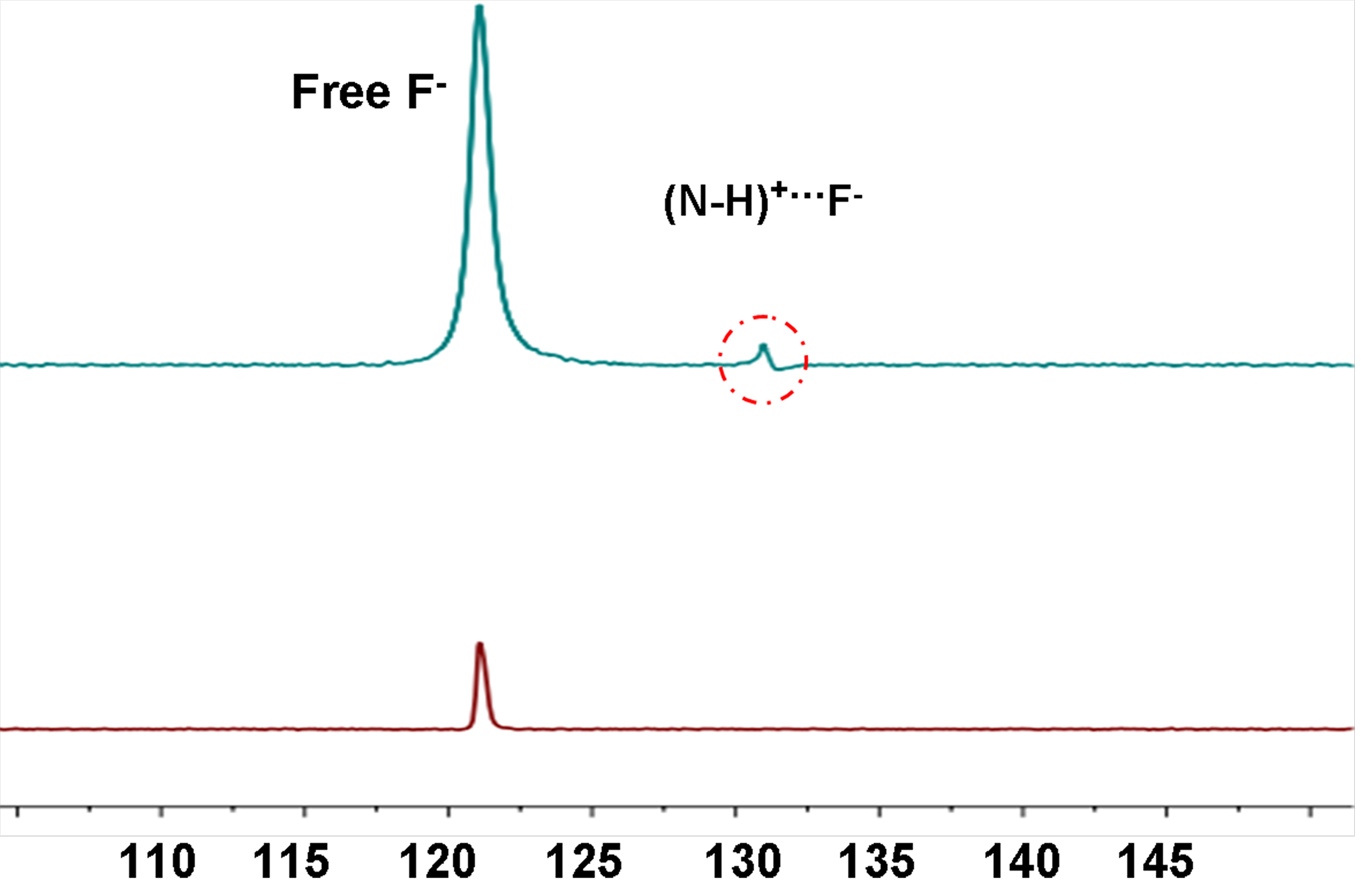

Supplement: Supplementary file 1 [file SC-006-C5SC02191J-s001.zip › fig_s5.tif]

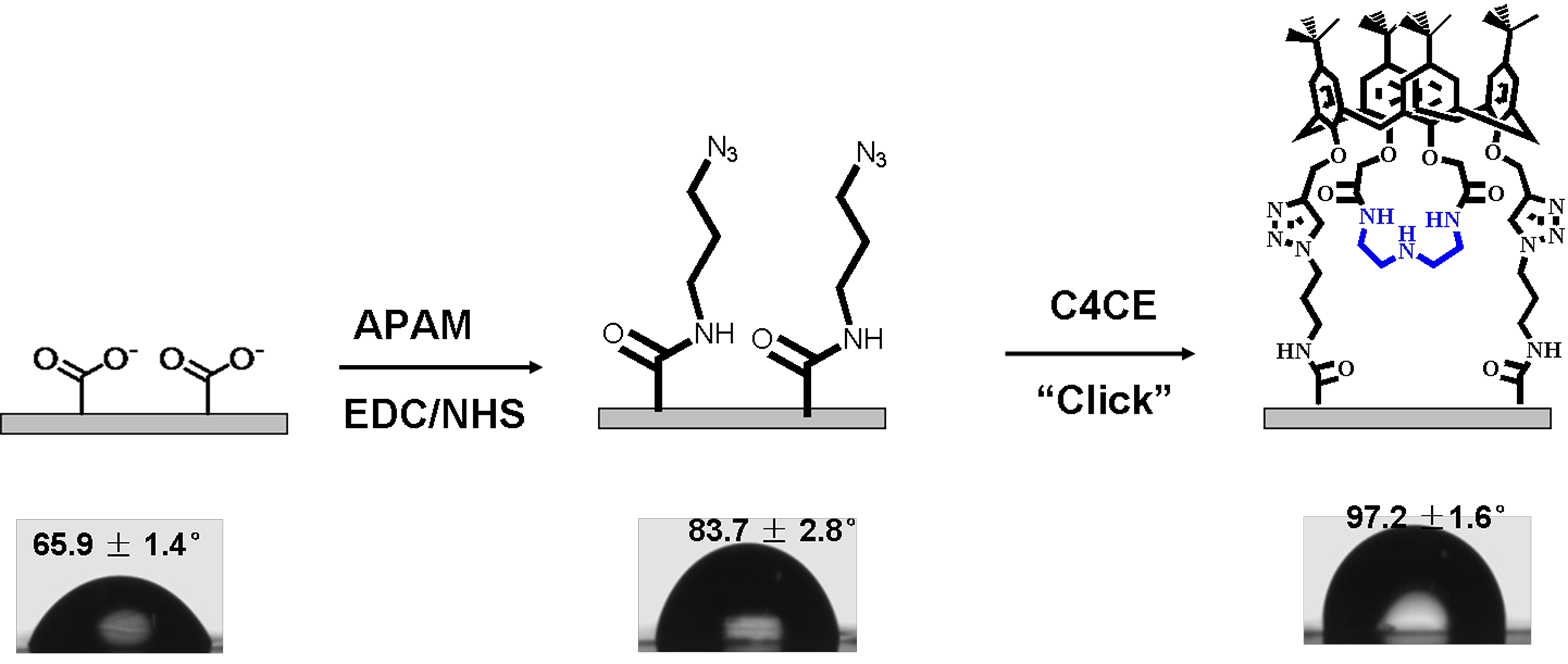

Supplement: Supplementary file 1 [file SC-006-C5SC02191J-s001.zip › fig_s6.tif]

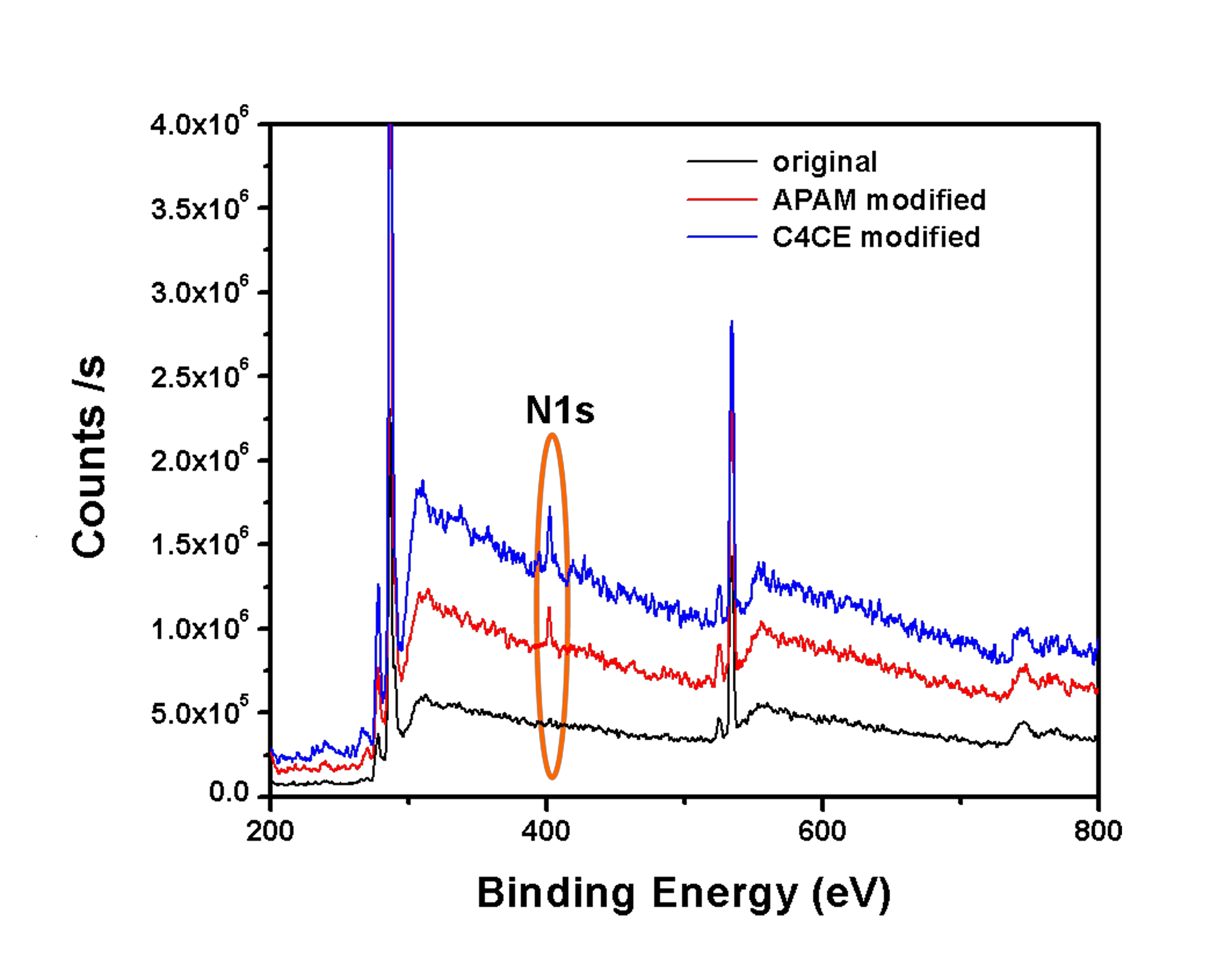

Supplement: Supplementary file 1 [file SC-006-C5SC02191J-s001.zip › fig_s7.tif]

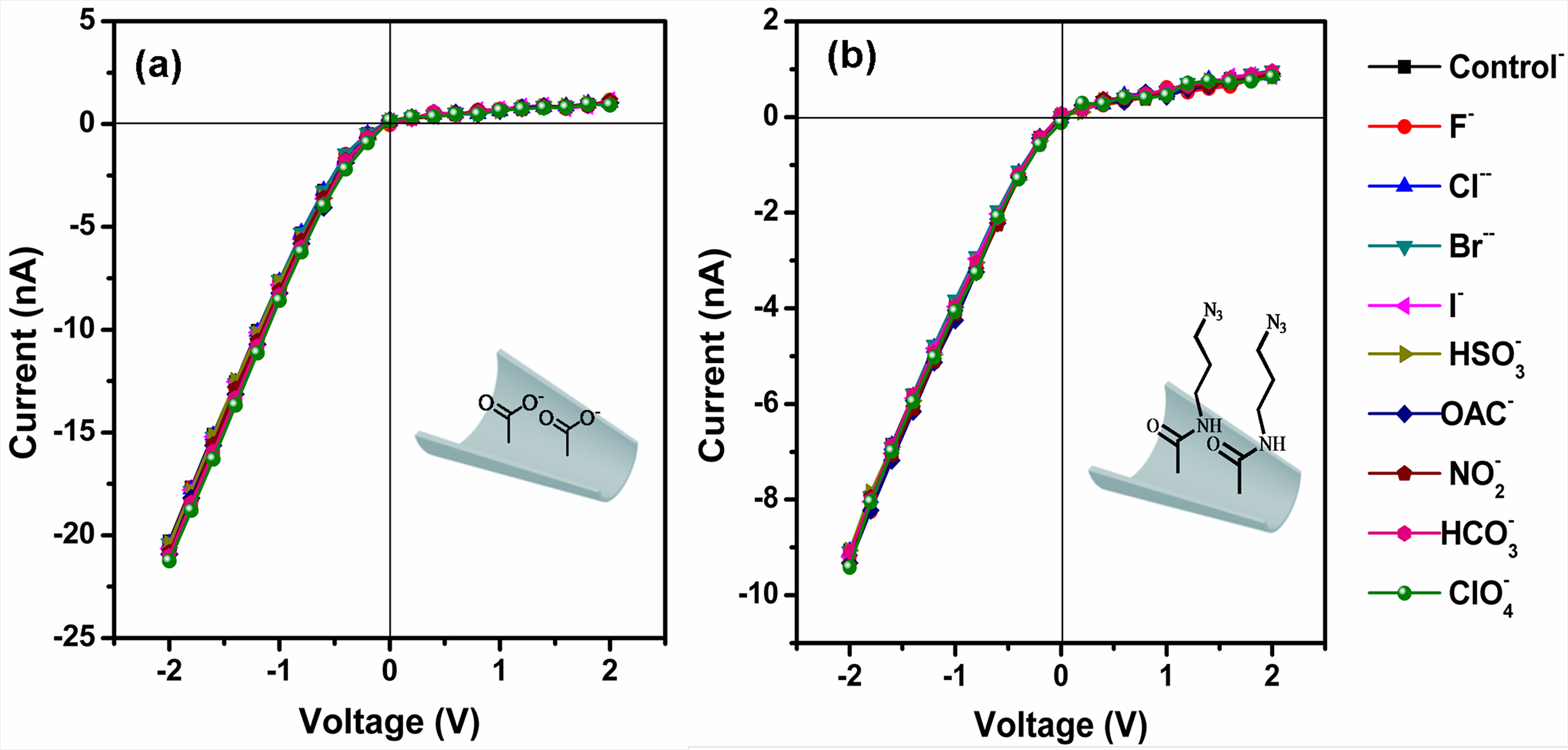

Supplement: Supplementary file 1 [file SC-006-C5SC02191J-s001.zip › fig_s8.tif]
