# Supplementary material for: Fluoride responsive single nanochannel: click fabrication and highly selective sensing in aqueous solution
Source: Chem Sci. 2015 Jul 22;6(10):5859–65. doi: 10.1039/c5sc02191j (PMC5950555; doi:10.1039/c5sc02191j)
Supplement: Supplementary file 2 [file SC-006-C5SC02191J-s002.pdf]

Electronic Supplementary Material (ESI) for Chemical Science.
This journal is © The Royal Society of Chemistry 2015

Supporting Information

Fluoride Responsive Single Nanochannel : Click Fabrication and Highly Selective Sensing in Aqueous Solution †

Guanrong Nie,[a] Yue Sun,[a] Fan Zhang [a],Miaomiao Song,[a] Demei Tian,[a] Lei Jiang[b] and Haibing Li *[a]

a Key Laboratory of Pesticide and Chemical Biology (CCNU), Ministry of Education, College of Chemistry, Central China Normal University, Wuhan 430079, (P.R. China)
E-mail: lhbing@mail.ccnu.edu.cn

b Beijing National Laboratory for Molecular Sciences (BNLMS) Key Laboratory of Organic Solids, Institute of Chemistry Chinese Academy of Sciences Beijing, 100190 (P.R. China)

Supporting Information

Contents

1. The synthesis and of the C4CE S3

2. ^{1}H NMR spectra of the C4CE (Fig S1) S4

3. ^{13}C NMR spectra of the C4CE (Fig S2) S5

4. ^{1}H NMR of the host C4CE and F^{-} (Fig S3) S6

5. ^{1}H NMR titration of the host C4CE and F^{-} (Fig S4) S7

6. ^{19}F NMR of the host C4CE and F^{-} (Fig S5) S8

7. Contact angles measurement (Fig S6)........ S9

8. X-ray photoelectron spectra (Fig S7, Table S1,S2,S3) S10

9. Laser scanning confocal microscopy S12

10. The *I-V* change for selectivity in original channel and APAM channel (Fig S8) S13

11. The *I-V* change in C4CE channel adding different concentration of F^{-} (Fig S9)........ S14

12. The linear fit by Langmuir equation (Fig S10) S15

13. The anti-interference performance (Table S4, Fig S11) S16

14. The calculation of the limit of detection (Table S5)........ S18

The synthesis of the C4CE

The synthesis of 1,3-dipropyl alkynyl-2,4-diester calix[4]arene (C4DE): 1, 3-dipropylalkynyl calix[4]arene (C4DY, 0.5 g, 0.69 mmol) was dissolved in dry THF (50 mL) and then NaH (0.66 g, 2.75 mmol) was added in batches. After stirring for 15 min, ethyl 2-bromoacetate (0.23 mL, 2.75 mmol) was added and the system was not stirred at room temperature until the raw material was run out of by the monitor of TLC for about 24 h. The product was obtained in white power through extraction and purified by ethyl acetate/ petroleum ether(v/v = 1:20) . The yield was 80%.

The synthesis of C4CE: the 1,3-dipropyl alkynyl-2,4-diester calix[4]arene (0.1 g, 0.11 mmol) was dissolved in 20 mL of dry methylbenzene/ methyl alcohol(v/v = 1:1) and then N'-(2-aminoethyl)ethane-1,2-diamine (0.05 mL, 0.33 mmol) was added. the system was not stirred at room temperature until the raw material was disappeared by the monitor of TLC for about 72 h. The white product with the yield of 65% was obtained by re-crystallization in chloroform/ methanol.

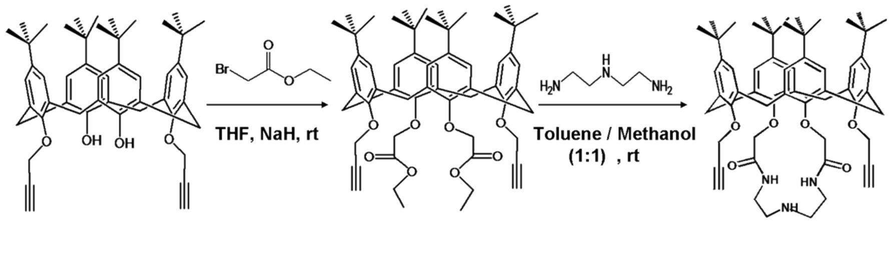

Scheme 1： the synthesis route of the host C4CE.

Spectra analysis of C4CE. ^{1}H NMR (DMSO): δ: 7.64(s, 2H, CONH), 7.22 (s, 4H, ArH), 6.49 (s, 4H, ArH), 5.07 (s, 4H, OCH_2), 4.38 (d, J = 12.6 Hz, 4H, $ArCH_2Ar$), 4.08 (s, 4H, OCH_2), 3.54 (s, 4H, NCH_2CH_2N), 3.19 (d, J = 12.7 Hz, 4H, $ArCH_2Ar$), 2.80 (s, 4H, NCH_2CH_2N), 2.50(s, 2H, ≡CH), 1.31 (s, 18H, Bu^t), 0.78 (s, 18H, Bu^t); ^{13}C NMR (DMSO-d_6): δ: 167.08, 151.15, 149.42, 145.74, 144.39, 131.45, 125.38, 124.38, 80.62, 77.94, 73.58, 59.63, 50.10, 33.89, 33.13, 31.39, 30.46; MS: calcd for m/z = 907.5, found m/z = 930.8 ($M^+ Na^+$). Elemental analysis (Found: C 76.98; H 8.03; N 4.58; C4CE requires C 76.79; H 8.00; N 4.63%.)

¹H NMR spectra of the C4CE

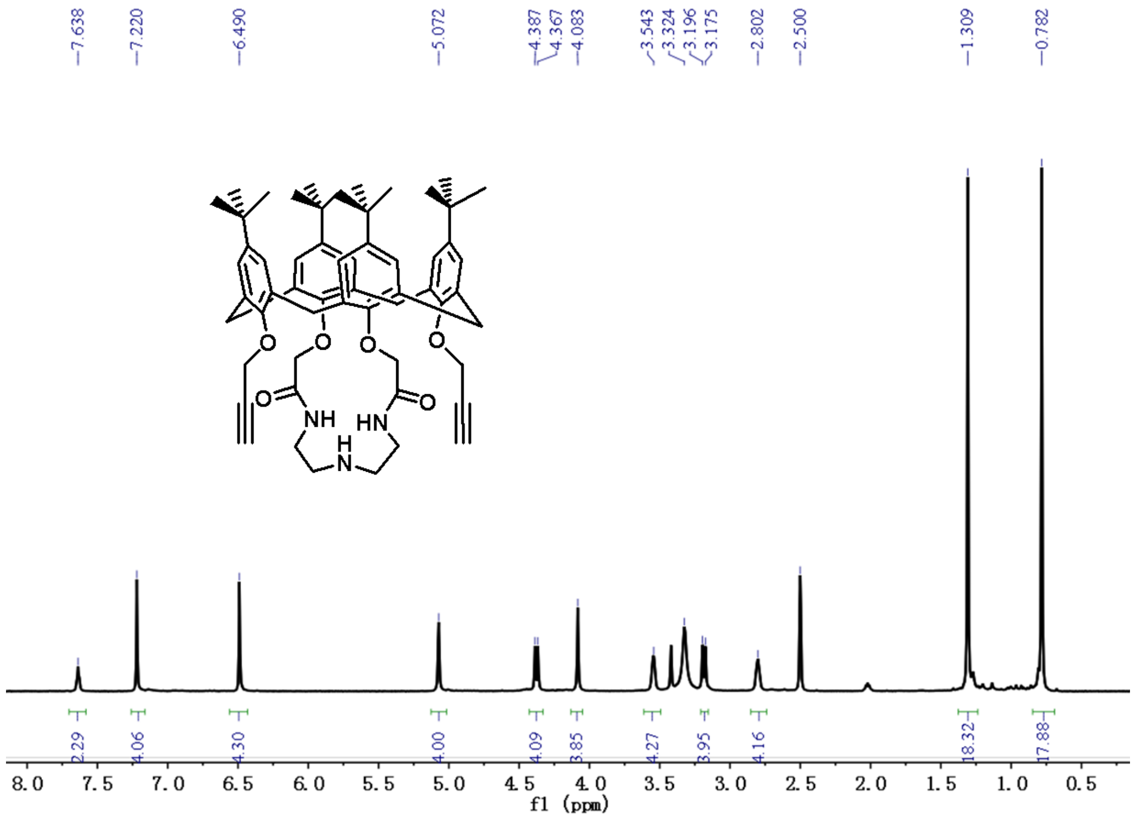

Fig S1 ¹H NMR spectra (DMSO) of the host C4CE.

^{13}C NRM spectra of the C4CE

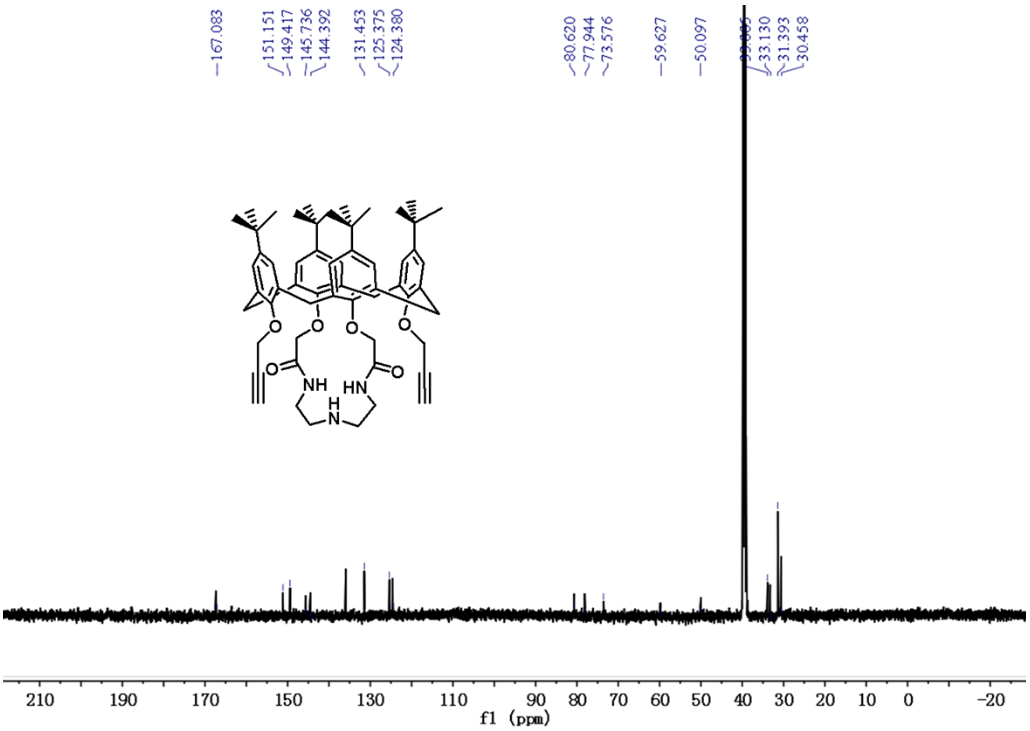

Fig S2: ^{13}C NMR spectra (100MHZ) of the host C4CE in DMSO-d_6.

^{1}H NMR of the host C4CE and F -

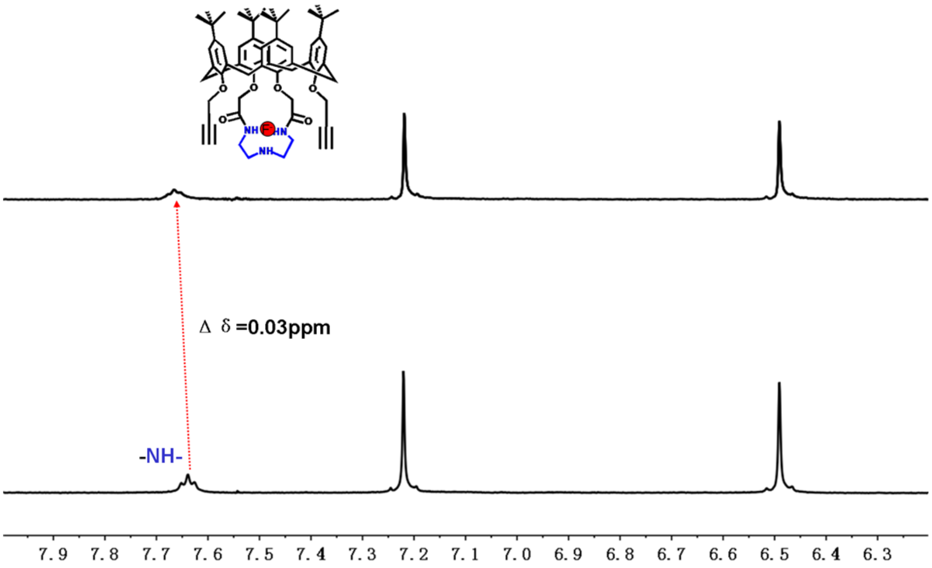

Fig S3 ^{1}H NMR spectra (DMSO, 400MHZ) of C4CE (2 mg) and 1 equivalent F^- . When the host and F^- was mixed, the –NH- proton peak of the host C4CE at 7.75 ppm is the downfield shift about 0.03 ppm after the F^- was added. The result was caused by the possibility of the hydrogen-bonding interaction of N-H···F.

^{1}H NMR titration of the host C4CE and F^-

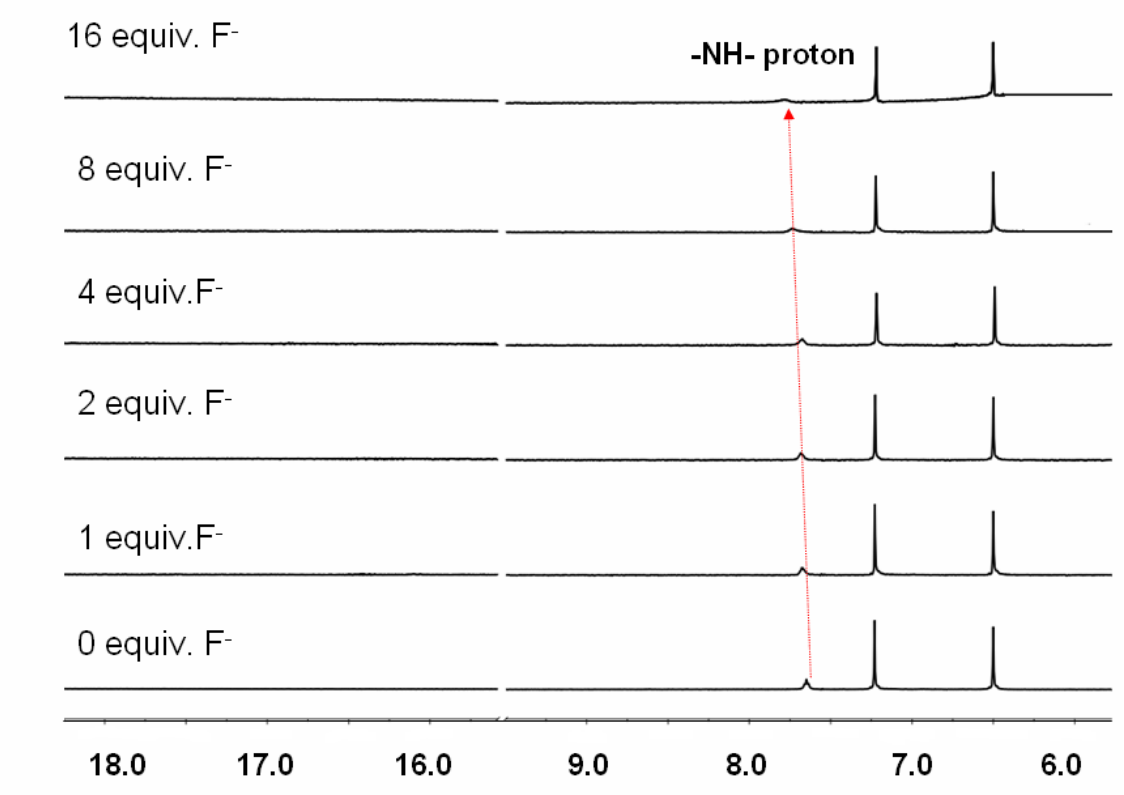

Fig S4 ^{1}H NMR titration spectra of host (2 mM) with 0, 1, 2, 4, 8, 16 equivalents of F^- ions in DMSO-d_6. The result indicated hydrogen-bonding interactions between the –NH- groups of the receptor with the F^- ions.

^{19}F NMR of the host C4CE and F^-

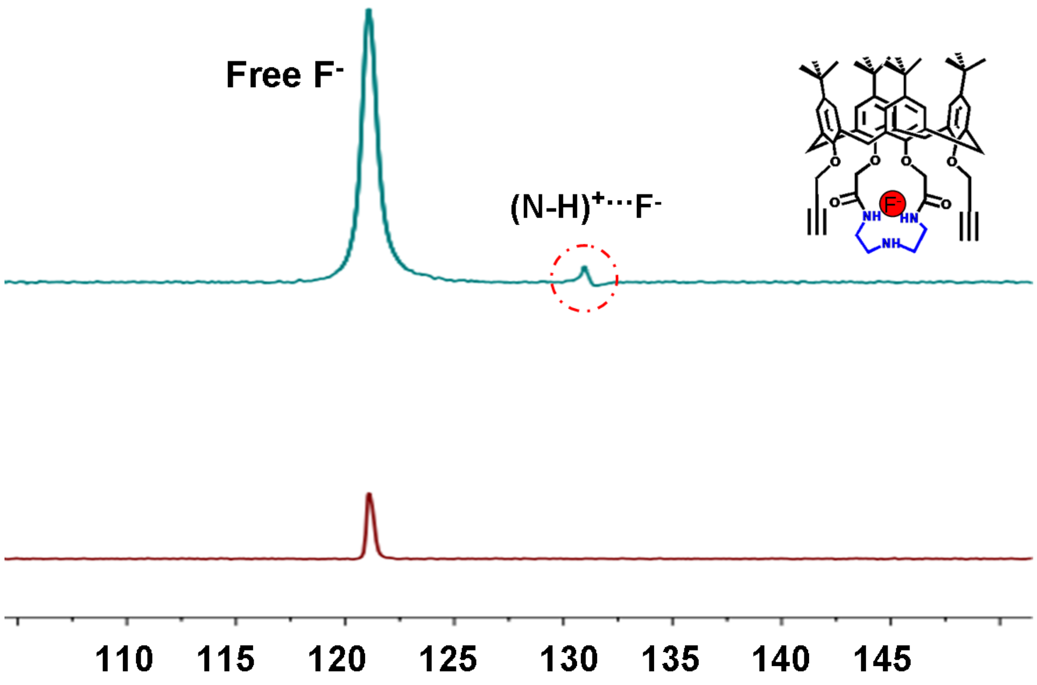

Fig S5 the ^{19}F NMR spectra of 5 mg of C4CE with 2 equivalent of F^- in 0.5 mL THF. The new peak appeared indicated the C4CE designed can interact with F^-

Contact angles measurement

Contact angles were measured using an OCA20 (DataPhysics, Germany) contact-angle system at ambient temperature and saturated humidity. The original PI membrane for contact angle measurement was treated with NaClO (13% available chlorine) at 50 ℃for 60 min. And then The sample was removed from the etching solution and treated with the stopping solution (1 M KI) for 20 min. After that, the sample was treated with distilled water overnight. Just as the modification method in the inner wall of the nanochannel 3-azidopropan-1-amine (APAM) and 1, 3-dipropargylaza-*p*-tert-butylcalix[4]crown (C4CE) was modified on the surface of the film successively. Before the contact angle test, the sample was blown dry with N_2. In each measurement, an about 1μL droplet of water was dispensed onto the surface of PI membrane. The average contact angel value was obtained at five different positions of the same membrane. As shown in Figure S5, the change of the wettability of the surface means the change of the chemical composition , to some extent, which indicated the successful modification of the APAM and C4CE.

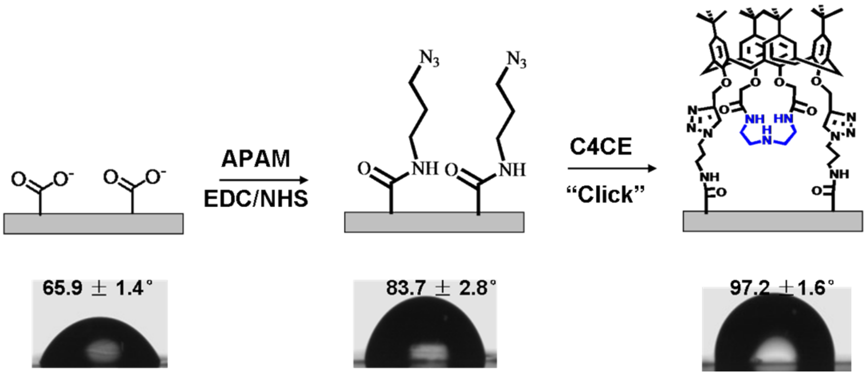

Fig S6 a) the contact angle without modification; b) the contact angle after the modification of 3-azidopropan-1-amine (APAM); c) the contact angle after the modification of 1, 3-dipropargylaza-*p*-tert-butylcalix[4]crown (C4CE) by click reaction. This suggests the C4CE was modified successfully by the click reaction.

XPS

X-ray photoelectron spectra (XPS) data were obtained with an ESCALab220i-XL electron spectrometer from VG Scientific using 300 W Al Kα radiation. In this work, to further prove the C4CE modified successfully by testing the nitrogen element, the PET film was used instead of the PI film. And also the COO^- was exposed after etching of the PET film, that was the same with the PI film. All peaks were referenced to C 1s (CHx) at 284.8eV in the deconvoluted high resolution C 1s spectra. The nitrogen element existed in the PET film indicates that 3-azidopropan-1-amine (APAM) and 1, 3-dipropargylaza-p-tert-butylcalix[4]crown (C4CE) was modified on the surface of the film successfully.

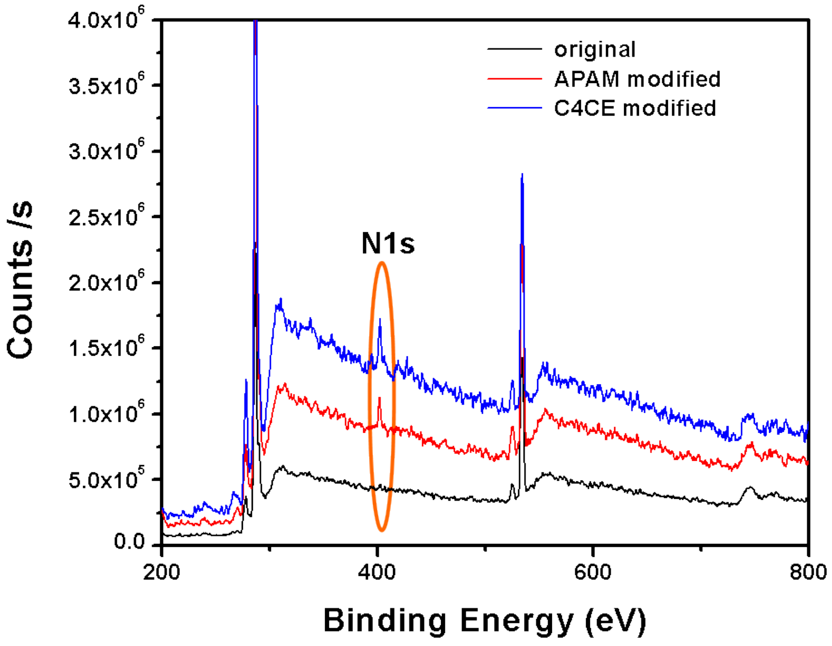

Fig S7 XPS spectra of PET films before and after modification. The control was referenced to the original film (black) . The modified APAM was referenced to the film after the modification of APAM (red), and the modified C4CE was referenced to the film after the modification of C4CE (blue). The nitrogen element existed in the PET film indicates that APAM and C4CE was modified on the surface of the film successfully.

Table S1 the XPS data of the film before modification

Name	Start BE	Peak BE	End BE	Height CPS	FWHM eV	Area (P) CPS.eV	At. %
C1s	295.12	284.83	281.68	20399.87	1.37	41268.73	71.38
O1s	537.92	531.83	526.52	17614.05	1.55	44187.69	28.62

Table S2 the XPS data of the film after APAM modification

Name	Start BE	Peak BE	End BE	Height CPS	FWHM eV	Area (P) CPS.eV	At. %
C1s	290.97	284.81	81.98	13704.49	1.31	27429.38	70.94
N1s	403.89	399.7	396.21	4516.56	1.51	9220.4	4.56
O1s	536.24	531.83	528.78	9892.76	1.64	25072.67	24.5

Table S3 the XPS data of the film after C4CE modification

Name	Start BE	Peak BE	End BE	Height CPS	FWHM eV	Area (P) CPS.eV	At. %
C1s	296.74	285.64	281.5	25299.59	2.07	69824.78	73.56
N1s	403.82	400.06	396.62	3735.4	2.15	10543.42	3.74
O1s	535.95	532.12	528.6	104032.3	2.01	235944.5	22.7

Laser scanning confocal microscopy

In order to directly characterize the F^- captured in the conical nanochannel, the host C4CE was derived and the dansyl chloride (DNS) severed as the fluorophore was introduced. According to the method in the literature, the DNS-C4CE was synthesis as follows: 1 mL of C4CE (1mM) in acetonitrile, 1 mL of DNS (2mM) in acetone and 2 mL of the buffer solution of sodium bicarbonate ($NaHCO_3$) at pH = 10.5 was added in flask. Subsequently, the flask was protected from the light for 1h. Fluorescence microscopic images were acquired by using a Zeiss confocal laser scanning unit mounted on a LSM710 fixed-stage upright microscope. In this experiment, the etched porous PI film was modified with the DNS-C4CE according to the same method in the single nanochannel. And then the DNS-C4CE -modified porous PI film was immersed in 10^{-3} M F^- solution for 3 h. Before the measurement, the film was washed by distilled water.

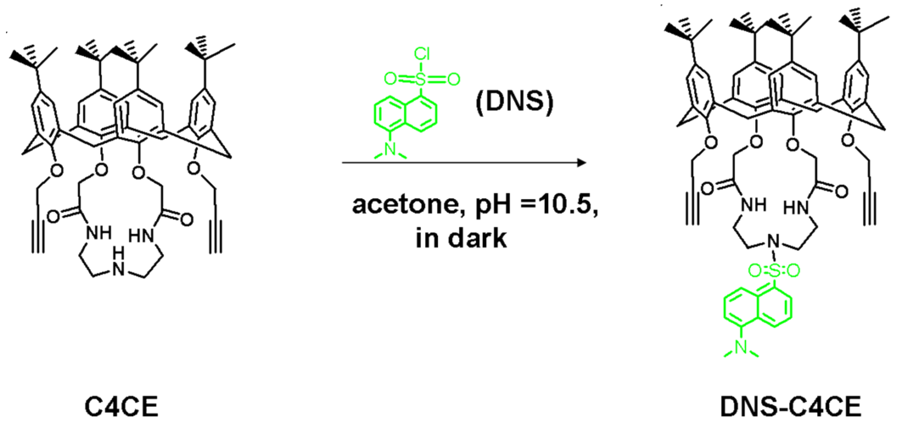

Scheme 2 The synthesis of DNS-C4CE with the fluorophore of dansyl chloride (DNS).

The I-V curve for selectivity in original channel and APAM channel

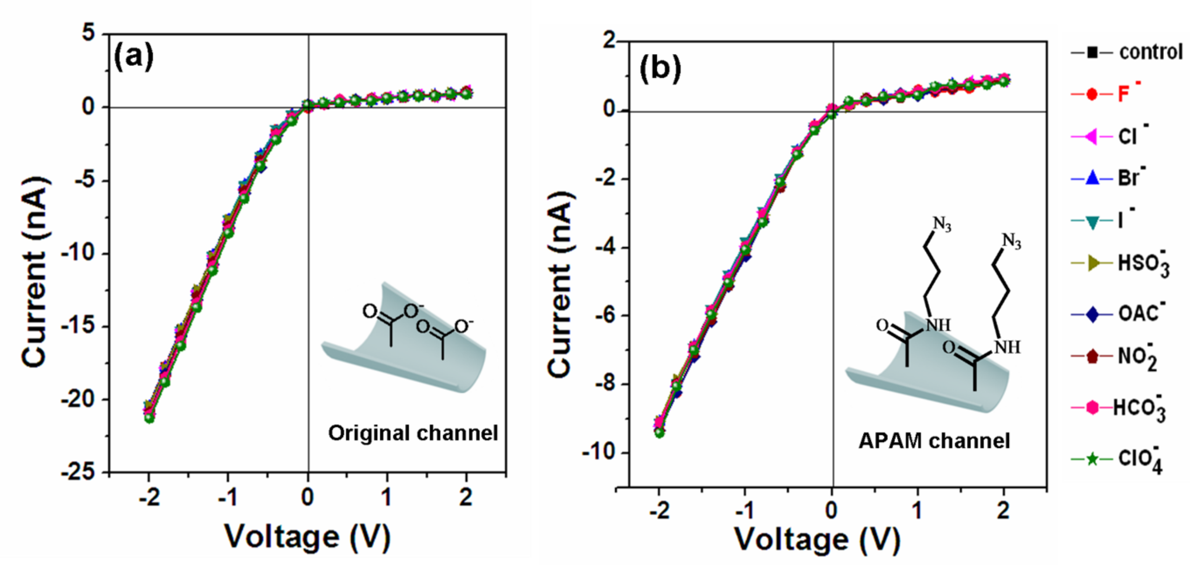

Fig S8 The *I-V* curve of the (a) original nanochannel and (b) APAM-modified nanochannel in the electrolyte adding 10^{-3} M F^-, Cl^-, Br^-, I^-, HSO_3^-, OAC^-, NO_2^-, HCO_3^-, ClO_4^-, respectively. No obvious current change indicated the original nanochannel and APAM-modified nanochannel was not selective for F^-.

The I-V curve in C4CE channel adding different concentration of F$^-$

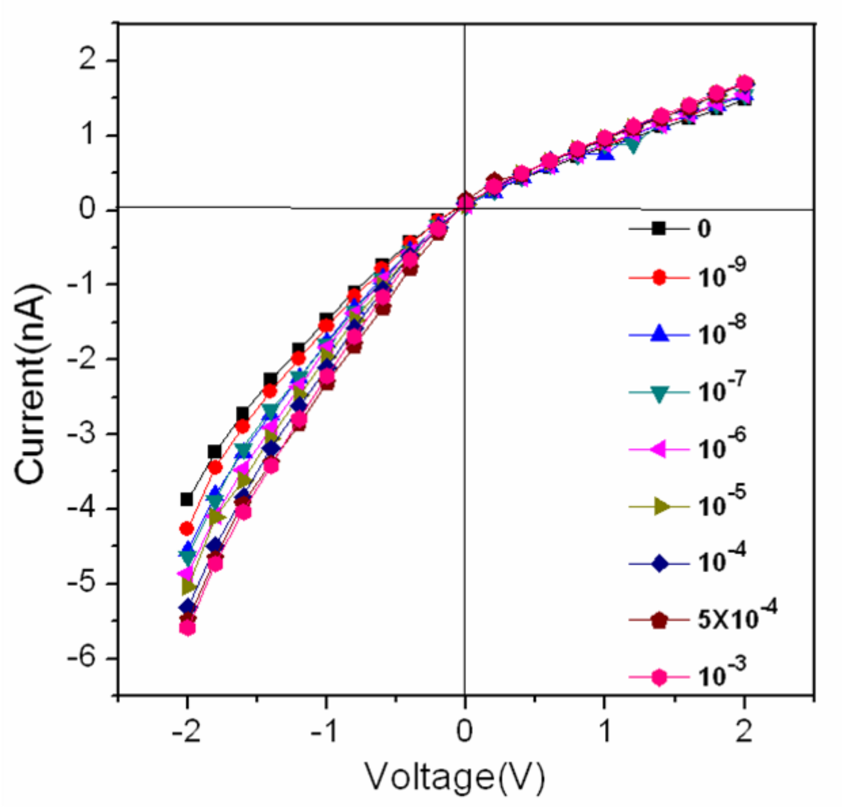

Fig S9 The *I-V* change of the nanochannel modified with C4CE in the presence of F$^-$ with the different concentration from 10^{-9} to 10^{-3} M

The linear fit by Langmuir equation

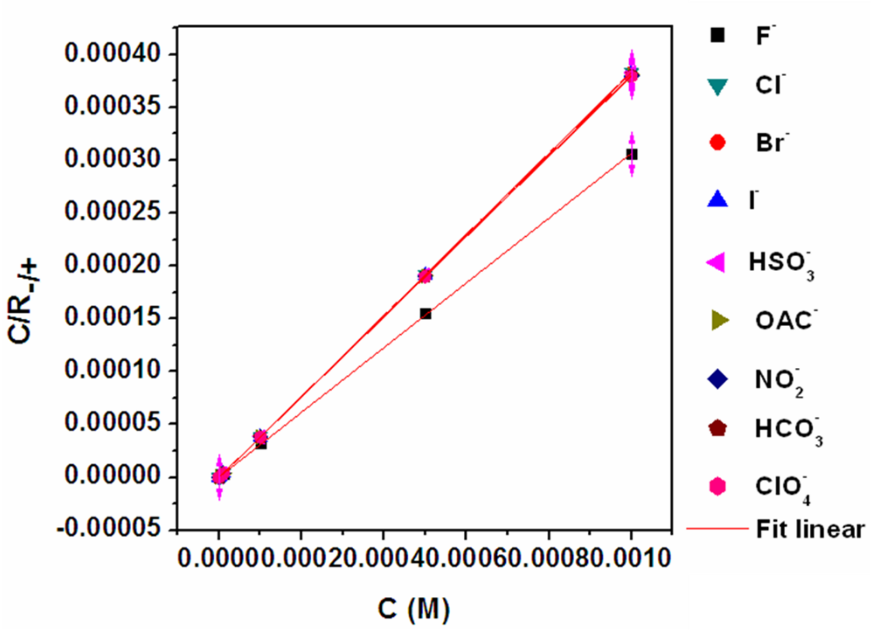

Fig S10 Langmuir isotherm plots of C4CE-modified nanochannel for the different ion adsorption and K (binding strength constant) was calculated. A Langmuir model ($C/R = 1/KR_{max} + C/R_{max}$), where C is the concentrations of ions, R is the rectification ratio ($R_{-/+} = I_{(-2V)} / I_{(2V)}$, the ratio of negative to positive current at -2 V), and K is the binding strength constant, was found to provide a perfect fit to the experimental data. Therefore, according to the date from Figure S8, $C/R_{-/+}$ was calculated and then the relation of $C/R_{-/+}$ — C was linear fit. All of the regression values (r^2) were above 0.9996. This different linears indicated the C4CE channel exhibited selectivity for F^- in quality.

The anti-interference performance

(1) the interference from the other anions

In this anti-interference test, the concentration of interfering ions added was 10^{-3} M and the concentration of determined F^- was 10^{-5} M. The individual interference ion and mixed interference ions was added and determined respectively. In Table 2, the Ratio denotes the relative ratio of the current change at -2 V and the expression was $(I-I_0) / I_0$, where I_0 was the current at -2 V in absence of the analytical ions and I was the current at -2 V in presence of the analytical ions. $Ratio_0$ was the value for F^- determination without interference ions and $Ratio_1$ was the value for F^- determination with interference ion. The recovery was calculated by the ratio of $Ratio_1$ and $Ratio_0$ to estimate the anti-interference performance.

Table S4: the recovery of F^- adding the different interfering ion respectively was calculated to evaluate the anti-interference performance

Interference	Interference ion/ $mol \cdot L^{-1}$	concentration of F^-/ $mol \cdot L^{-1}$	$Ratio_0$ without interference ion	$Ratio_1$ with interference ion	Recovery /%
Br^-	10^{-3}	10^{-5}	0.2860	0.3080	107.7
I^-	10^{-3}	10^{-5}	0.2860	0.3121	108.3
Cl^-	10^{-3}	10^{-5}	0.2860	0.3002	104.9
HSO_3^-	10^{-3}	10^{-5}	0.2860	0.2872	95.20
OAC^-	10^{-3}	10^{-5}	0.2860	0.3105	106.9
NO_2^-	10^{-3}	10^{-5}	0.2860	0.3283	109.7
HCO_3^-	10^{-3}	10^{-5}	0.2860	0.2857	90.20
ClO_4^-	10^{-3}	10^{-5}	0.2860	0.3221	95.40
Mixed ions	10^{-3}	10^{-5}	0.2860	0.3413	93.10

(2) the interference from the serum

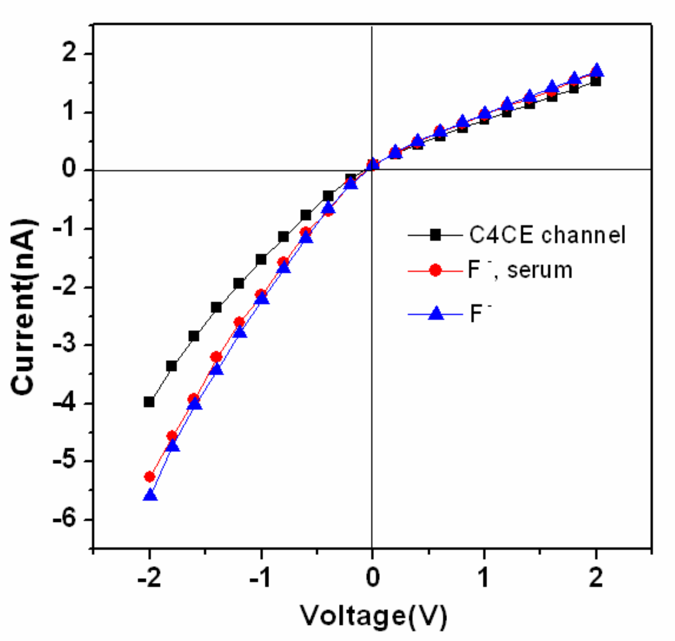

Fig S11 the *I-V* change was measured in the electrolyte of 0.1 M KCl-Tris-HCl (black line), 0.1 M KCl- Tris-HCl + 10^{-5} M F^- (blue line), 0.1 M KCl-Tris-HCl (pH = 7.0)+10^{-5} M F^- + serum (10^4 times diluted). The recovery calculated was 79.8%, which suggested the C4CE channel still exhibited good response to F^- in serum sample.

The calculation of the limit of detection

The C4CE channel was measured for 9 times in the pure electrolyte of 0.1 M KCl- Tris-HCl without adding any analytical ions and $R_{-/+}$ was obtained. According to the Linear equation of F^- by linear fit, the corresponding C was gained . And then the standard deviation was calculated by the formula of S= $\sqrt{\frac{\sum_{i=1}^{n}(C_i - \overline{C})}{n-1}}$. Therefore, the limit of detection (LOD=3S) was 9.30×10^{-7} M.

Table S5: the calculation of the limit of detection

Times	$R_{-/+}$	Linear equation of F^-	Corresponding C / M	standard deviation S= $\sqrt{\frac{\sum_{i=1}^{n}(C_i - \overline{C})}{n-1}}$	Limit of detection (LOD=3S) / M
1	2.48	$C/R_{-/+}=0.3063C+2.6101\times10^{-7}$	2.70×10^{-6}	3.10×10^{-7}	9.30×10^{-7}
2	2.51		2.86×10^{-6}		
3	2.60		3.30×10^{-6}		
4	2.60		3.36×10^{-6}		
5	2.56		3.24×10^{-6}		
6	2.60		3.35×10^{-6}		
7	2.46		2.62×10^{-6}		
8	2.49		2.72×10^{-6}		
9	2.58		3.14×10^{-6}		
